# Regulation of the *Fasciola hepatica* newly excysted juvenile cathepsin L3 (FhCL3) by its propeptide: a proposed ‘clamp-like’ mechanism of binding and inhibition

**DOI:** 10.1186/s12860-020-00335-5

**Published:** 2020-12-07

**Authors:** Izanara C. Pritsch, Irina G. Tikhonova, Heather L. Jewhurst, Orla Drysdale, Krystyna Cwiklinski, Marcelo B. Molento, John P. Dalton, Carolina De M. Verissimo

**Affiliations:** 1 Department of Basic Pathology, Federal University of Parana, Curitiba, 81531-970 Brazil; 2 School of Biological Sciences, Queen’s University Belfast, Belfast, Northern Ireland UK; 3 School of Pharmacy, Medical Biology Centre, Queen’s University Belfast, Belfast, BT9 7BL UK; 4 Centre for One Health and Ryan Institute, School of Natural Sciences, National University of Ireland Galway, Galway, Ireland; 5 Department of Veterinary Medicine, Federal University of Parana, Curitiba, Paraná, Brazil

**Keywords:** Cathepsin L, Cysteine peptidases, Propeptide, Inhibitor, *Fasciola hepatica*

## Abstract

**Background:**

The zoonotic worm parasite *Fasciola hepatica* secretes an abundance of cathepsin L peptidases that are associated with virulence, invasiveness, feeding and migration. The peptidases are produced as inactive zymogens that activate at low pH by autocatalytic removal of their N-terminal pro-domain or propeptide. Propeptides bind to their cognate enzyme with high specificity. Little is known, however, about the mechanism by which the propeptide of FhCL3, a cathepsin L peptidase secreted by the infective newly excysted juveniles (NEJs), regulates the inhibition and activation of the mature enzyme before it is secreted into host tissues.

**Results:**

Immunolocalisation/immunoblotting studies show that the FhCL3 zymogen is produced and secreted by gastrodermal cells of the NEJs gut. A recombinant propeptide of FhCL3 (ppFhCL3) was shown to be a highly potent and selective inhibitor of native and recombinant *F. hepatica* FhCL3 peptidase, and other members of the cathepsin L family; inhibition constant (*K*_*i*_) values obtained for FhCL1, FhCL2 and FhCL3 were 0.04 nM, 0.004 nM and < 0.002 nM, respectively. These values are at least 1000-fold lower than those *K*_*i*_ obtained for human cathepsin L (HsCL) and human cathepsin K (HsCK) demonstrating the selectivity of the ppFhCL3 for parasite cathepsins L. By exploiting 3-D structural data we identified key molecular interactions in the specific binding between the ppFhCL3 and FhCL3 mature domain. Using recombinant variants of ppFhCL3 we demonstrated the critical importance of a pair of propeptide residues (Tyr^46^Lys^47^) for the interaction with the propeptide binding loop (PBL) of the mature enzyme and other residues (Leu^66^ and Glu^68^) that allow the propeptide to block the active site.

**Conclusions:**

The FhCL3 peptidase involved in host invasion by *F. hepatica* is produced as a zymogen in the NEJs gut. Regulation of its activation involves specific binding sites within the propeptide that are interdependent and act as a “clamp-like” mechanism of inhibition. These interactions are disrupted by the low pH of the NEJs gut to initiate autocatalytic activation. Our enzyme kinetics data demonstrates high potency and selectivity of the ppFhCL3 for its cognate FhCL3 enzyme, information that could be utilised to design inhibitors of parasite cathepsin L peptidases.

**Supplementary Information:**

The online version contains supplementary material available at 10.1186/s12860-020-00335-5.

## Background

*Fasciola hepatica* is a global parasite of humans and their livestock (sheep, cattle and water buffalo) [1, 2]. The parasite has the widest latitudinal and longitudinal distribution of any worm parasite, largely because of its ability to infect and complete its life cycle in a wide range of mammalian hosts. Part of the parasite’s wide-ranging virulence is attributed to the expression and secretion of large amounts of papain-like cysteine peptidases, namely cathepsin L and cathepsin B [3]. Within the parasite genome, these peptidases have expanded and evolved into multi-membered families through a process of gene duplication and diversification that has generated an array of hydrolases with overlapping and distinct, and sometimes unique, substrate specificities [4, 5]. The strict differential expression of these peptidases in juvenile and adult stages of *F. hepatica* reinforces the idea that the parasite synchronises their expression and secretion to match the obstacles that each developmental stage encounters, and must overcome, within its host [3, 6, 7].

Infection of the host is dependent on the secretion of a specific set of these peptidases. Following ingestion of the encysted infective stage of *F. hepatica* (metacercariae), the parasites emerge in the intestine as newly excysted juveniles (NEJs). NEJs immediately secrete cathepsin L peptidases, named FhCL3, and cathepsin B peptidases, termed FhCB1, FhCB2 and FhCB3, into the tissues; these are the most abundant proteins found within the in vitro secretome of this life cycle stage [7–9]. By a combination of forward mechanical pressure and hydrolytic tissue degradation the parasite rapidly traverses the intestinal wall to enter the peritoneum. FhCL3 has been of particular interest because of its remarkable ability to digest native type I and II collagen, which allows the NEJs to disrupt the extracellular matrix of tissues and facilitates their penetration through the intestinal wall [4, 10, 11].

Precise regulation of peptidase activity is essential for host-parasite interaction. The *F. hepatica* cathepsin L peptidases are secreted as inactive pro-enzymes or zymogens. An N-terminal extension or ‘propeptide’ sits on the surface of the mature active enzyme in an extended conformation and runs through the active site cleft in the opposite direction to a protein substrate, thereby preventing peptidase activity [12, 13]. The cathepsin L propeptides are ~ 100 amino acid in length (~ 12 kDa) and contain two conserved motifs, ERFNIN and GNFD, which are thought to mediate interactions with the mature cathepsin domain [13–15]. Studies on mammalian cathepsin L peptidases show that the propeptides act as chaperones that are essential for correct folding and cellular trafficking of the mature domain [13]. However, their primary role is in the regulation of peptidase activity, acting as reversible competitive inhibitors [15] as thus preventing premature activation of the zymogen to a mature enzyme that could cause uncontrolled cellular or tissue damage [12, 13]. Cathepsin L peptidase activation occurs by bimolecular autocatalytic cleavage of the propeptide at its juncture with the mature domain that is triggered by an acidic pH and/or by trans-processing by another active peptidase such as asparaginyl endopeptidase [5, 13, 16, 17].

Since the N-terminal cathepsin propeptide of cysteine peptidases are highly specific and potent regulators of their cognate enzyme [13, 18], they can act as useful structural templates to design active-site directed, selective, and efficient inhibitors of cathepsin peptidases [19–22]. Uncovering how propeptides bind to their cognate mature domain is, therefore, not only fundamental to understanding parasite virulence and infection, but also important in gaining information that could be exploited for anti-parasite drug design.

Considering the dearth of information surrounding the selectivity among the propeptides of the *F. hepatica* cathepsin L family, in this study we investigated the mechanism involved in FhCL3 inhibition/interaction by its propeptide (ppFhCL3). We chose to investigate FhCL3 because it is indispensable in the infection process and exhibits unique collagenolytic activity. Using a recombinant ppFhCL3, we show that this is a highly potent and selective inhibitor of the mature enzyme, FhCL3. 3-D structural data identified key molecular interactions between the propeptide and mature enzyme. We validated their importance in propeptide binding by producing ppFhCL3 variants and determining their inhibitory kinetics against several parasite and human cathepsin L peptidases. Thus, we pinpointed the paired propeptide residues Tyr^46^Lys^47^, which bind to the propeptide binding loop (PBL) in the mature domain, and the residues Leu^66^ and Glu^68^, which bind within the active site cleft, as critical to propeptide binding potency and specificity. From our studies, we propose that these binding sites are interdependent and act as a “clamp-like” mechanism of inhibition. Our results offer new insights on propeptide-enzyme interactions that could be utilised in the design of effective inhibitors of cathepsin L peptidases.

## Results

### FhCL3 zymogen and propeptide co-localise within the NEJs gastrodermis

**Fig. 1:**
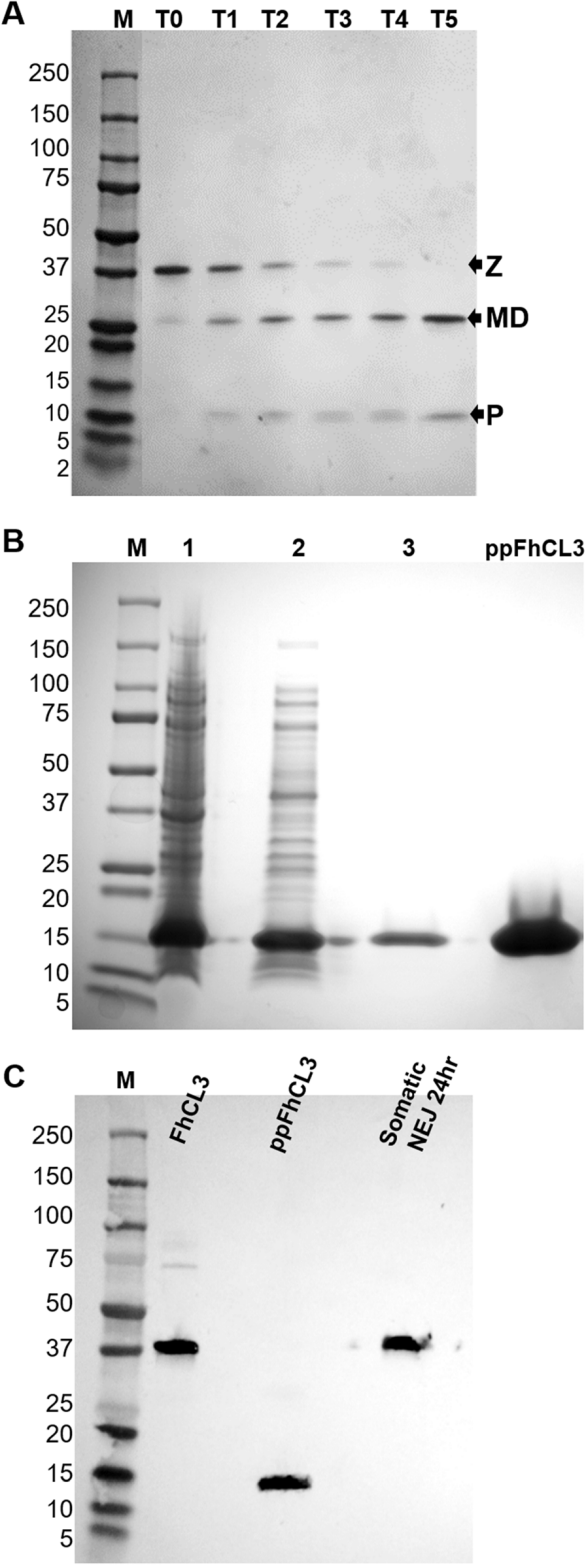
Production of functional recombinant FhCL3 zymogen and FhCL3 propeptide. (**a**) SDS-PAGE gel showing the activation of the recombinant FhCL3 zymogen. T0: Inactivated recombinant FhCL3 zymogen expressed in the yeast *P. pastoris* and purified using affinity chromatography. T1 – T5: Activation of the FhCL3 zymogen was initiated by incubating the enzyme in sodium acetate buffer pH 4.5 at 37 °C for 5 h. The progression of activation was observed by removing samples at each hour, adding the inhibitor E-64 before resolving in a 4–12% SDS-PAGE gel. Z, zymogen; MD, mature domain of FhCL3; P, the released propeptide. (**b**) SDS-PAGE gel showing expression of the recombinant FhCL3 propeptide in *E. coli* BL21 cells. Lane 1, cell pellet after induction for 3 h at 30 °C; lane 2, supernatant after extraction of the cell pellet; lane 3, wash after recombinant protein binding to the affinity column (Profinia affinity chromatography system); lane 4, the eluted recombinant ppFhCL3. (**c**) Western blot analysis of recombinant FhCL3 zymogen (1 μg), FhCL3 propeptide (2 μg), and somatic extract of NEJs 24 h (15 μg). Immunoblots were probed with anti-ppFhCL3 polyclonal antibodies raised in rabbit. M, Molecular weight in kDa

A functional recombinant 37 kDa FhCL3 zymogen was expressed in the methylotrophic yeast *Pichia pastoris* and purified by affinity chromatography. The enzyme could be autocatalytically activated in vitro by incubation at pH 4.5 for 5 h brought about by the cleavage of the N-terminal propeptide (~ 12 kDa) from the mature enzyme domain (~ 25 kDa) (Fig. 1a and Additional file 5).

Recombinant ppFhCL3 was expressed in *Escherichia coli* B21 cells and isolated by affinity chromatography as a soluble protein of the expected molecular size of ~ 12 kDa (Fig. 1b). Western-blot analysis showed that polyclonal antibodies prepared against the ppFhCL3 bound to the 37 kDa recombinant zymogen and, as expected, to the 12 kDa recombinant ppFhCL3. The antibodies also recognised the 37 kDa native FhCL3 zymogen in somatic extracts of NEJs. Notably, the polyclonal antibodies prepared against the recombinant FhCL3 zymogen were demonstrated to be predominantly directed against the mature domain of the FhCL3 (see Additional file 6).

**Fig. 2:**
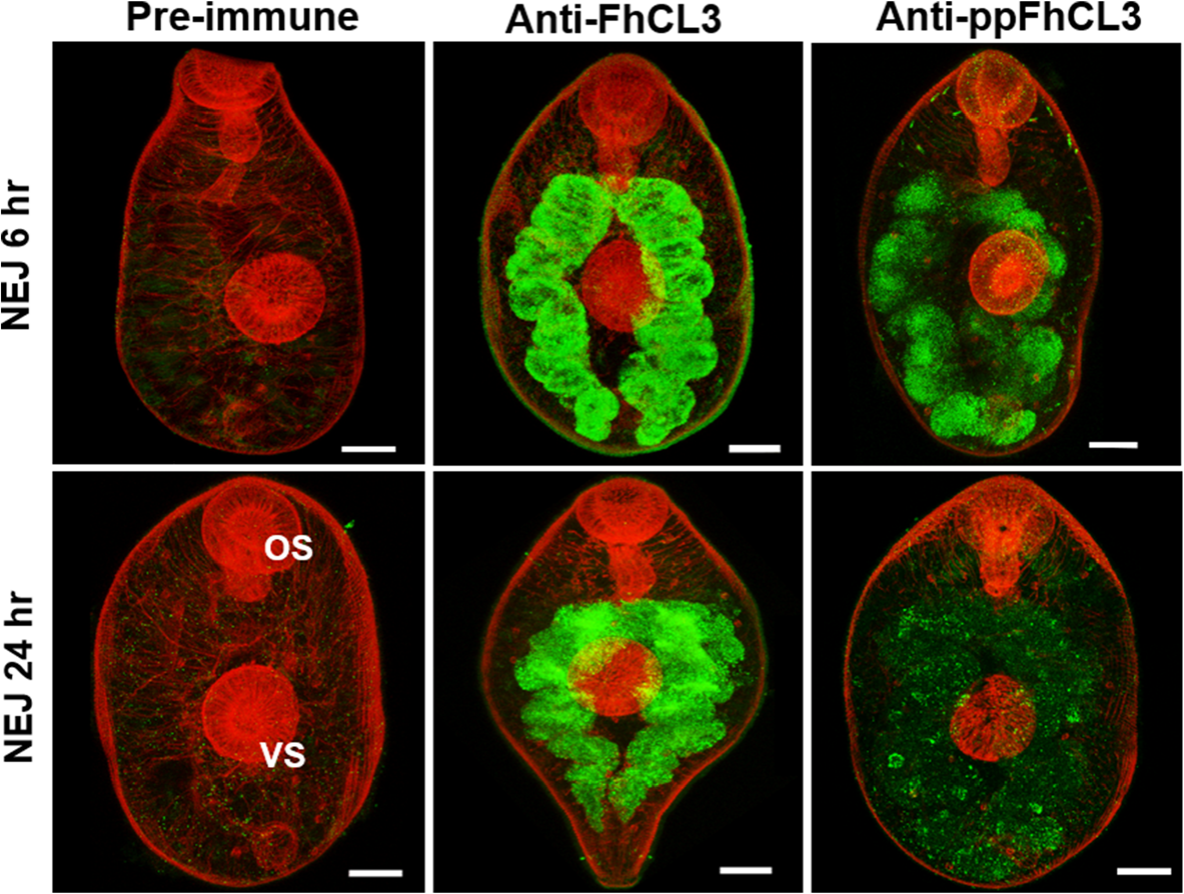
Immunolocalisation of FhCL3 zymogen and FhCL3 propeptide within *F. hepatica* NEJs. NEJs cultured for 6 h and 24 h post-excystment were fixed with 4% paraformaldehyde, washed and then probed with pre-immune rabbit antiserum, anti-FhCL3 zymogen polyclonal antibodies raised in rabbit, or anti-ppFhCL3 polyclonal antibodies raised in rabbit. Immunolocalisation of proteins was visualised using a secondary antibody fluorescein isothiocyanate (FITC)-labelled goat anti-rabbit IgG (green fluorescence). Counter-staining with phalloidin-tetramethylrhodamine isothiocyanate (TRITC) provided the background structure (red fluorescence). OS: oral sucker; VS: ventral sucker. Scale bars, 20 μM

Antibodies prepared against the FhCL3 zymogen and ppFhCL3 were used to probe whole paraformaldehyde-fixed *F. hepatica* NEJs obtained at 6 h and 24 h post-excystment, which demonstrated that both FhCL3 zymogen and propeptide localise within the bifurcated gastrodermis of the parasite (Fig. 2). The immunolocalisation using the anti-FhCL3 zymogen antibody resulted in a fluorescent signal consistently stronger than the signal obtained with anti-ppFhCL3 antibody, a difference we found was even more pronounced in 24 h NEJs (Fig. 2b and c).

### FhCL3 propeptide differentially inhibits *F. hepatica* and human cathepsin peptidases

We characterised the ppFhCL3 binding activity and selectivity against a range of recombinant *F. hepatica* cathepsin L peptidases, namely FhCL1, FhCL2 and FhCL3, and against human cathepsin L (HsCL) and human cathepsin K (HsCK) peptidases over a pH range 4.5 to 7.0. At pH 7.0, 10 nM of ppFhCL3 inhibited ~ 100% of the activity of all three recombinant *F. hepatica* cathepsin L peptidases. Inhibition of FhCL1, FhCL2 and FhCL3 was optimal at pH 7.0 and decreased as the pH was decreased, whereby at pH 4.5 the propeptide exhibited no inhibition. The ppFhCL3 did not significantly inhibit the *F. hepatica* cathepsin B peptidases (see Additional file 1).

**Fig. 3:**
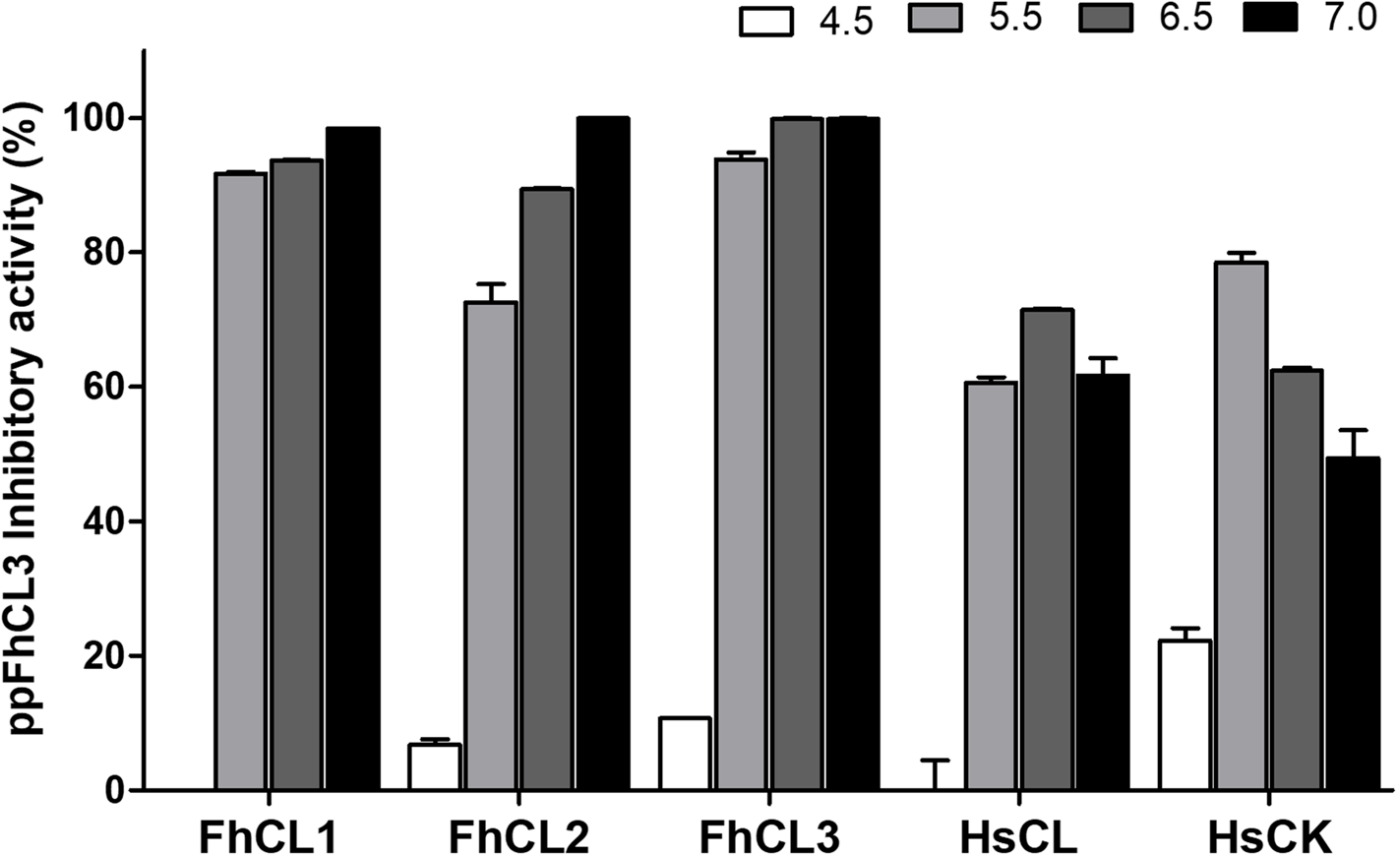
Effect of pH on the inhibitory activity of the ppFhCL3 against cathepsin peptidases. The activity of the recombinant *F. hepatica* cathepsin peptidases, FhCL1, FhCL2, FhCL3, and human cathepsin L and K, HsCL and HsCK, was assayed in the presence of ppFhCL3 (10 nM) at various pH, 4.5–7.0, represented by the white, grey-scale and black bars. The percentage inhibition is presented relative to the total activity of each enzyme without inhibitor at the respective pH. The experiments were performed in triplicate and the results are presented as mean ± standard deviation

The propeptide was less efficient against the human cathepsin peptidases; the optimum inhibition towards HsCL (~ 70%) and HsCK (~ 80%) was obtained at pH 6.5 and 5.5, respectively, and while no inhibition against HsCL was observed at pH 4.5 some activity against HsCK was detected (~ 20%) (Fig. 3). Finally, ppFhCL3 showed no activity against the serine peptidases, trypsin, chymotrypsin, thrombin or kallikrein (see Additional file 1).

### FhCL3 propeptide binds and inhibits native *F. hepatica* cathepsin L1, L2 and L5

**Fig. 4:**
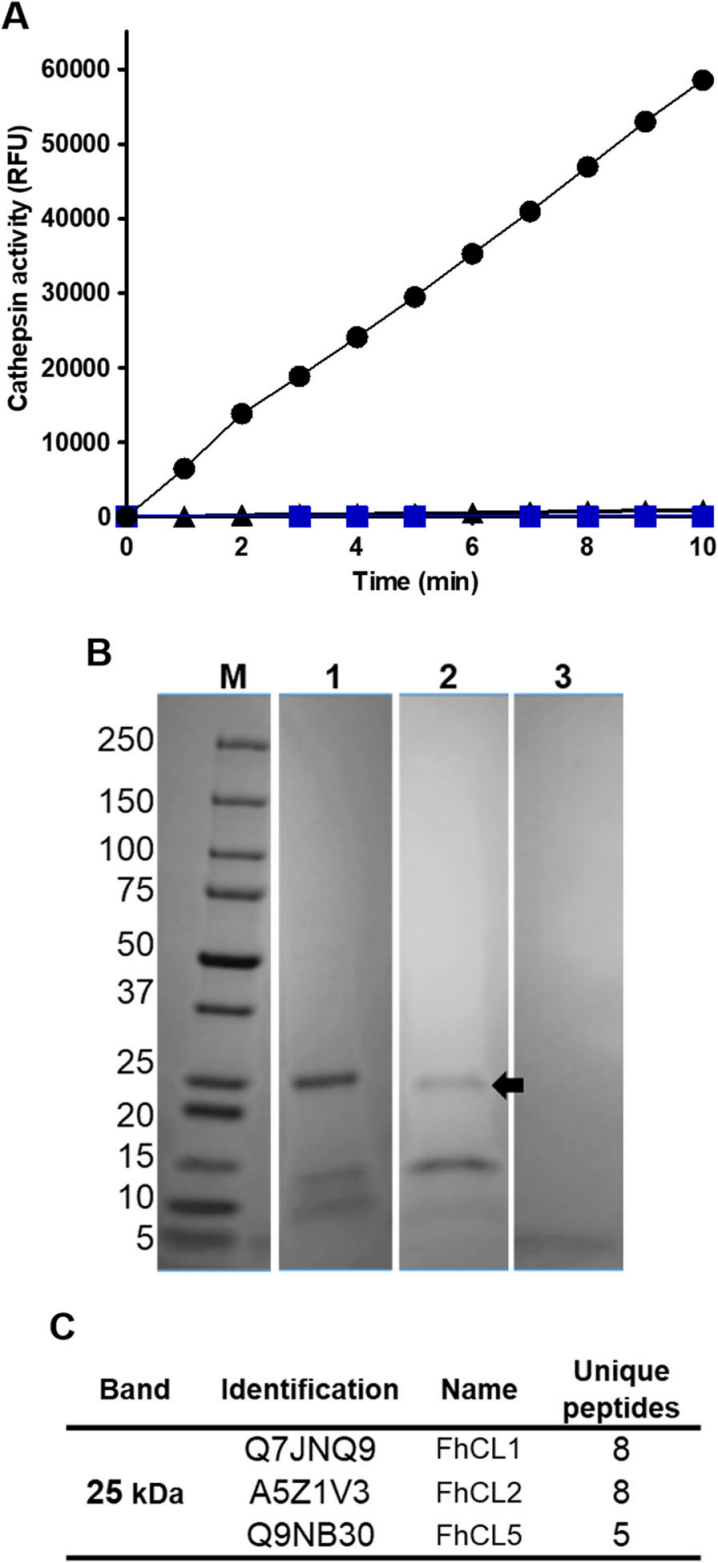
FhCL3 propeptide binds and inhibits native cathepsin peptidases excreted-secreted by *F. hepatica* adult parasites. (**a**) Cathepsin peptidase activity, presented as relative fluorescent units, in the excretory-secretory (ES) proteins from *F. hepatica* adult worms measured in the absence of the inhibitors (circles), in the presence of 10 nM recombinant ppFhCL3 (triangles) or in the presence of the broad-spectrum cysteine peptidases inhibitor, E-64 (blue squares). (**b**) ppFhCL3 was used to pull-down proteins within the ES proteins from adult *F. hepatica* and the results were analysed by SDS-PAGE gels, as follows: Lane 1, ES proteins from *F. hepatica* adult parasite; Lane 2, ES proteins from *F. hepatica* adult parasites pulled down with Ni-NTA beads/recombinant ppFhCL3; Lane 3, ES proteins from *F. hepatica* adult parasites pulled down with Ni-NTA beads; M, molecular weight in kDa. (**c**) Mass-spectrometry analyses (LC-MS/MS) of the proteins in the ~ 25 kDa band of the Fig. 4B (arrow)

Previous proteomic studies have shown that the composition of the molecules secreted from *F. hepatica* adult parasites in vitro (excretory-secretory products/proteins; ES products) is dominated by cathepsin L peptidases FhCL1, FhCL2 and FhCL5 (> 80% of total protein) [5]. When the total peptidase activity in the adult ES products was assayed, using the cathepsin L fluorogenic peptide Z-Leu-Arg-NHMec as a substrate, it showed a linear reaction over 10 min (Fig. 4a). However, addition of the recombinant ppFhCL3 (10 nM) to the reaction completely abrogated the enzymatic activity in the ES products (Fig. 4a). This inhibition was as strong as that obtained with the broad-spectrum cysteine protease inhibitor, E-64, which confirmed that the hydrolytic activity in adult ES products is derived from cysteine peptidases (Fig. 4a).

In order to identify the ES proteins with which the ppFhCL3 was interacting, a pull-down experiment was performed. The recombinant ppFhCL3 was first attached to Ni-NTA beads via the histidine tag before adding to the adult parasite ES products. Those proteins that attached to the propeptide-beads were pulled-down by centrifugation and subsequently resolved by SDS-PAGE (Fig. 4b). A ~ 25 kDa band, consistent with the molecular size of mature cathepsin L peptidases, was observed in the pull-down (Fig. 4b, lane 2). LC-MS/MS analysis of the ~ 25 kDa band identified the three different adult *F. hepatica* cathepsin L peptidases, namely FhCL1, FhCL2 and FhCL5. This data shows that the FhCL3 propeptide can bind several cathepsin L peptidases expressed by *F. hepatica* other than FhCL3 (Fig. 4b)*.*

### Structural studies highlight key interactions between the FhCL3 propeptide and the mature enzyme domain

**Fig. 5:**
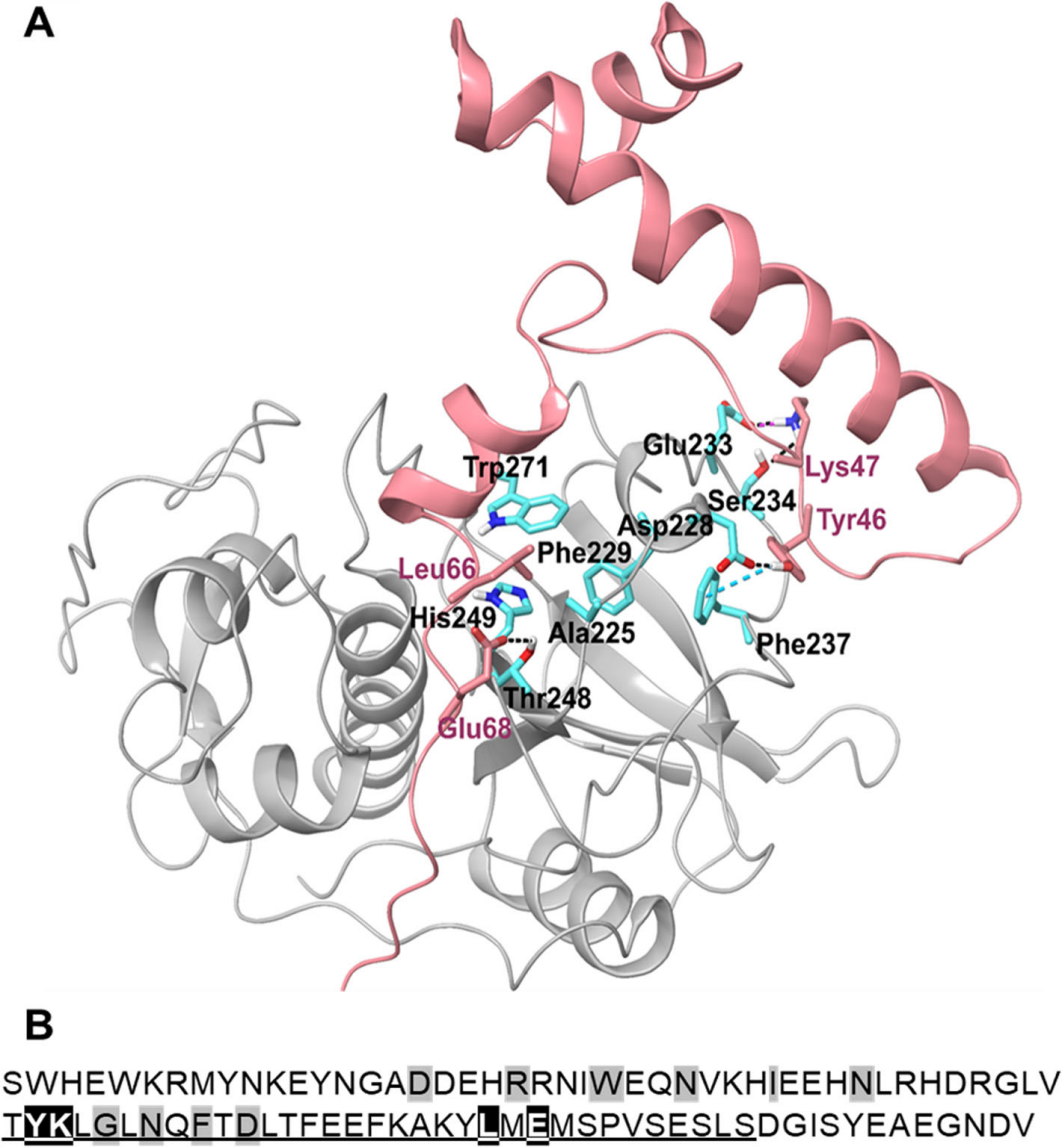
Structural representation of the FhCL3 zymogen highlighting the interaction of the propeptide with the mature enzyme. (**a**) The 3-D modelling of FhCL3 zymogen suggests the importance of key ppFhCL3 amino acid residues (pink) that stabilize the propeptide binding/inhibition (in pink) with the FhCL3 mature domain (grey; residues and labels in cyan and black). The ppFhCL3 residue Tyr^46^ binds to Asp^228^ and Phe^237^, while Lys^47^ binds to Ser^234^ and Glu^233^ residues within the propeptide binding loop (PBL), respectively. The C-terminal portion of the propeptide is then inserted into the substrate binding cleft, and specific interaction between the propeptide residue Leu^66^ with the hydrophobic pocket of Ala^225^, Phe^229^, His^249^ and Trp^271^, and Glu^68^ with Thr^248^ in the mature enzyme result in a tight binding that prevent substrate entrance in the enzyme cleft. Dotted lines represent hydrogen bonds (black), a salt bridge (pink) and π-π interaction (cyan) formed between residues of the propeptide and the mature enzyme. (**b**) Primary amino acid sequence of the ppFhCL3. Residues highlighted in black are predicted to be involved in fundamental interactions with the mature enzyme domain to stabilize the binding. The sequence portion of the ppFhCL3 used as a template to produce a synthetic peptide (33 residues) is underlined. Residues highlighted in grey form the propeptide conserved motifs ERFNIN and GNFD

A structural model of the FhCL3 zymogen was obtained by homology modelling using the crystal structure of the FhCL1 zymogen as a template previously resolved by us [12] (Fig. 5a). The sequence identity between FhCL1 and FhCL3 is high, 71% indicating a reliable 3D-model could be constructed. The FhCL3-ppFhCL3 complex was modelled based on the complex of the human cathepsin L bound to a propeptide as a starting point and optimized using molecular dynamics simulations. This in silico analysis provided insights into the propeptide-mature enzyme interactions. Consistent with all cathepsin L peptidases [13], the N-terminal portion of the propeptide is primarily alpha-helical in structure and presses against the mature enzyme domain. The 3-D model revealed that this interaction is fastened by a pair of residues, p46-tyrosine (Tyr^46^) and p47-lysine (Lys^47^). In particular, Tyr^46^ forms a hydrogen bond with Asp^228^ and π-π-interaction with Phe^237^, whereas Lys^47^ forms a salt bridge with Glu^233^ and a hydrogen bond with Ser^234^. The second major interaction we observed between the propeptide and mature domain occurs where the propeptide penetrates the active site cleft in the reverse direction to a normal protein substrate. Two propeptide residues, p66-Leucine (Leu^66^) and p68-Glutamic acid (Glu^68^), make key interactions that secure the propeptide inside the active site and prevents substrate entry (Fig. 5a). Thus, the Leu^66^ sits in the hydrophobic pocket composed of Ala^255^, Phe^229^, His^249^ and Trp^271^ while the Glu^68^ forms a hydrogen bond with Thr^248^.

Since we showed that ppFhCL3 also inhibited the major cathepsin L peptidases of *F. hepatica,* FhCL1, FhCL2 and FhCL5, as well as HsCL and HsCK, we mapped the corresponding residues present in the mature domains of the different cathepsin peptidases that are likely involved in interacting with ppFhCL3 (Table 1). This comparison showed that there is substantial variation between the residues situated at these binding positions, which may explain the difference in binding kinetics observed for the ppFhCL3 with these peptidases (see below).

**Table 1:** FhCL3 propeptide amino acid residues predicted to interact with the propeptide binding loop (PBL) and substrate binding site of the mature *F. hepatica* and human cathepsin L and cathepsin K peptidases

ppFhCL3	Residues within the mature cathepsin				
	FhCL3	FhCL2	FhCL1	HsCL	HsCK
Tyr^46^	Asp^228^ ^a^Phe^237^	Asp^248^	Asp^248^	Ser^255^	Ser^255^
Lys^47^	^b^Ser^234^ ^c^Glu^233^	Ser^254^	Ser^254^	Glu^261^ Lys^260^	Lys^261^ Ser^260^
Leu^66 *^	Ala^225^	Ala^245^	Val^245^	Gly^252^	Ser^252^
Glu^68 *^	Thr^248^	Thr^268^	Asn^268^	Asp^275^	Asn^275^

* Residues interacting within the substrate binding site of the cathepsin peptidase

a, π-π interaction; b, hydrogen bonds; c, salt bridge formed between residues of the ppFhCL3 and the mature domain of FhCL3

### Substitution of key residues within the propeptide demonstrate their importance in binding to the mature cathepsin domain

The importance of the interactions between the ppFhCL3 residues Tyr^46^, Lys^47^, Leu^66^ and Glu^68^ in binding and inactivation of the mature enzyme domain was investigated by producing three recombinant FhCL3 propeptide variants containing substitutions designed to de-stabilise these interactions (Fig. 5b). These variants were as follows: (a) ppFhCL3_Tyr^46^Lys^47^/Ala^46^Ala^47^, in which the paired Tyr^46^ and Lys^47^ were each replaced by the hydrophobic residue alanine; (b) ppFhCL3_Leu^66^/Gly^66^, whereby Leu^66^ was substituted by the small amino acid glycine; and (c) ppFhCL3_Glu^68^/Arg^68^, whereby the negatively charged Glu^68^ was replaced by a positive charged arginine residue.

**Fig. 6:**
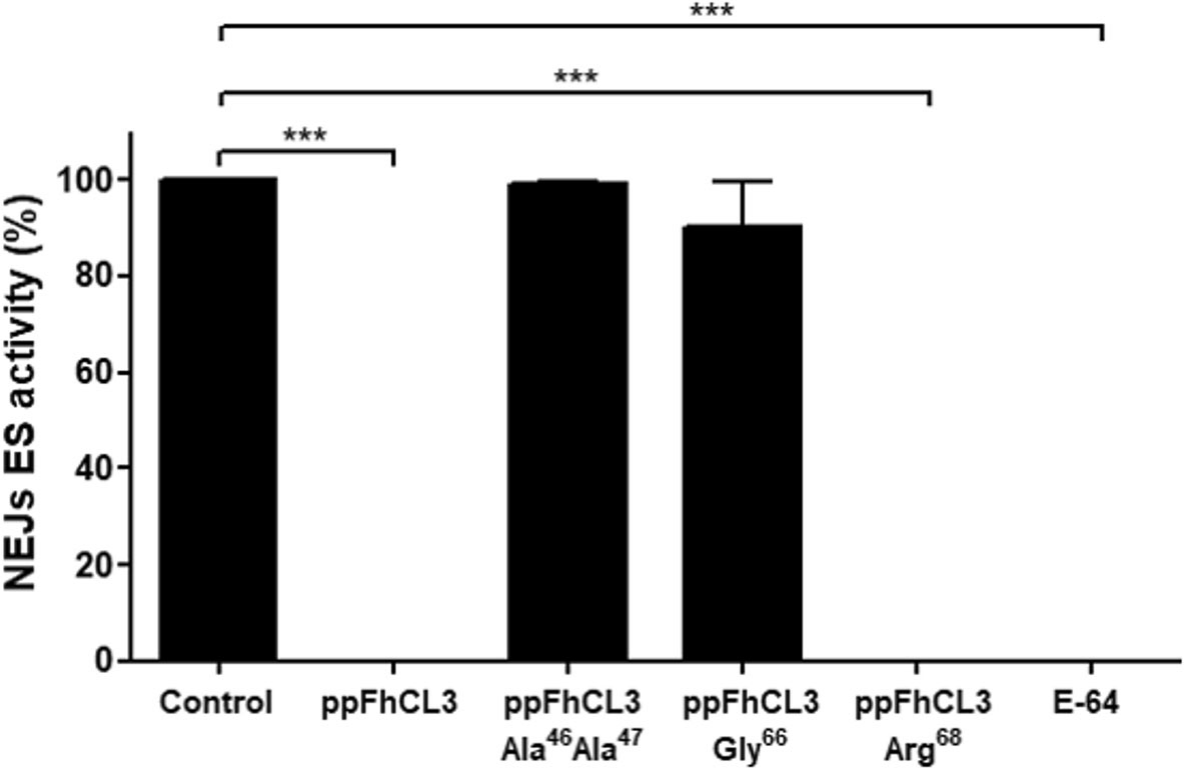
Inhibitory activity of the wild-type and variant FhCL3 propeptides against cathepsin peptidases excreted-secreted by *F. hepatica* NEJs. The inhibitory activity of the wild-type ppFhCL3 (10 nM), variant proteins ppFhCL3_Tyr^46^Lys^47^/Ala^46^Ala^47^ (10 nM), ppFhCL3_Leu^66^/Gly^66^ (10 nM), ppFhCL3_Glu^68^/Arg^68^ (10 nM) or the broad-spectrum cathepsin inhibitor E-64 were assayed against the cathepsin peptidases excreted-secreted by NEJs 24 h post-excystment. The percentage of inhibition was calculated relative to the activity within the NEJs ES alone (Control). The experiments were performed in triplicate and the results are presented as mean ± standard deviation. Statistical analysis was carried out using One-way ANOVA with Dunnett multiple comparison (*** *P ≤* 0.001)

After recombinant production and purification (see Additional file 2), the propeptide variants were first tested against native cathepsin peptidases in the ES products from NEJs 24 h post-excystment, which proteomic studies have demonstrated contains predominantly FhCL3 [5]. We used the substrate Z-Gly-Pro-Arg-NHMec to specifically assay for FhCL3 (Table 3). Our data show that the wild-type ppFhCL3 (10 nM) completely inhibited the cathepsin L peptidase activity in the NEJs ES products. Similarly, total inhibition was also observed with the variant ppFhCL3_Glu^68^/Arg^68^; however, by contrast, the variant ppFhCL3_Leu^66^/Gly^66^ and the dual variant ppFhCL3_Tyr^46^Lys^47^/Ala^46^Ala^47^ did not inhibit the peptidase activity in the ES products (Fig. 6).

**Fig. 7:**
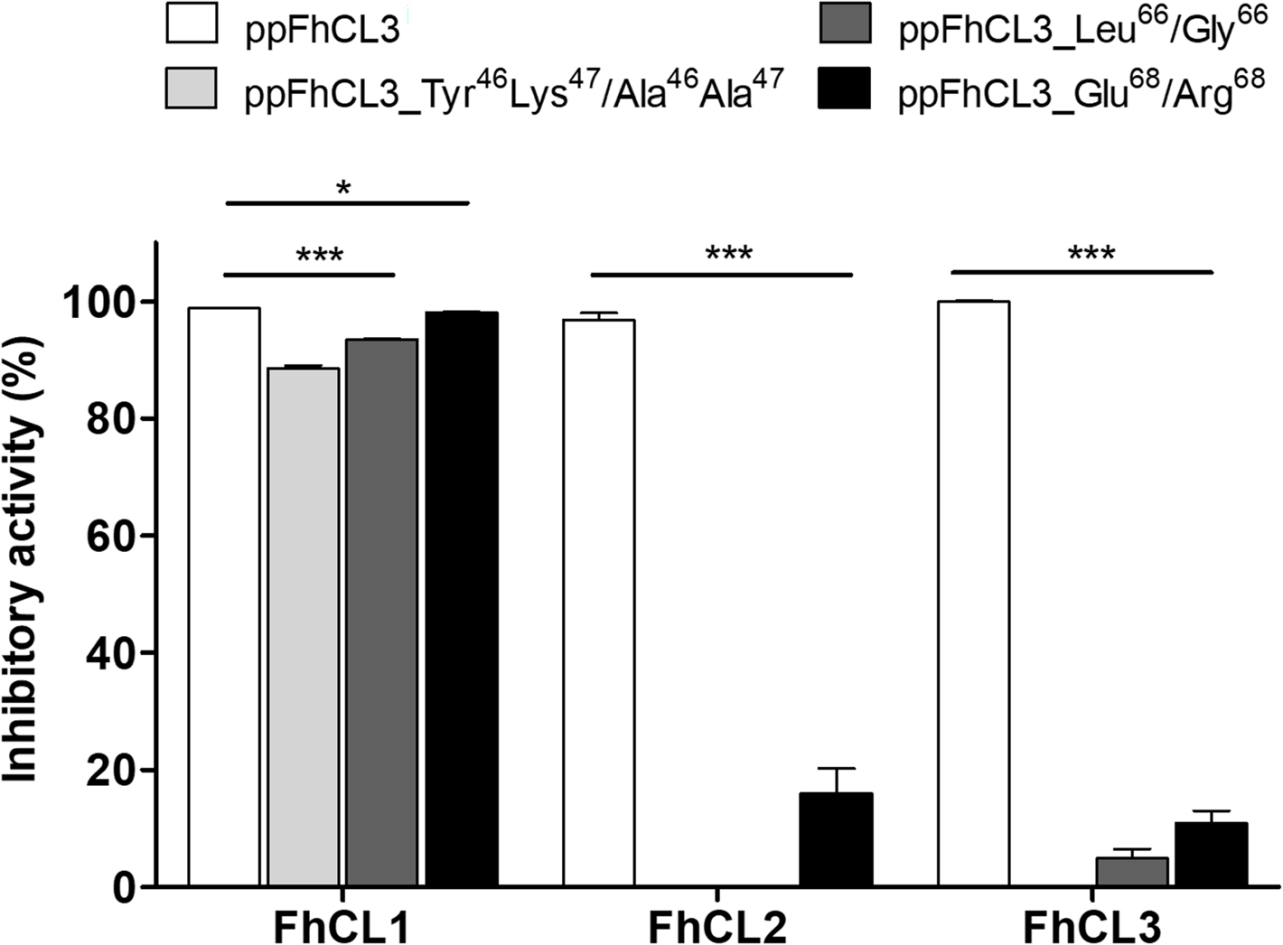
Inhibition profile of wild-type and variant FhCL3 propeptides against recombinant *F. hepatica* cathepsin L peptidases. The inhibitory activity of wild-type ppFhCL3 and variant ppFhCL3_Tyr^46^Lys^47^/Ala^46^Ala^47^, ppFhCL3_Leu^66^/Gly^66^ and ppFhCL3_Glu^68^/Arg^68^ was examined at 10 nM against recombinant cathepsin L peptidases FhCL1, FhCL2 and FhCL3. The inhibitory activities are presented relative to the total activity of each enzyme alone. The experiments were performed in triplicate and the results are presented as mean ± standard deviation. Statistical analysis was carried out using One-way ANOVA with Dunnett multiple comparison (* *P* *<* 0.05, *** *P ≤* 0.001)

We further explored the inhibitory profile of the wild-type ppFhCL3 and its variants against recombinant *F. hepatica* cathepsin L peptidases, FhCL1, FhCL2 and FhCL3. Consistent with our previous results, 10 nM of wild-type ppFhCL3 inhibited FhCL1, FhCL2 and FhCL3 activity by ~ 98%, ~ 97% and ~ 100%, respectively. Surprisingly, at this concentration, all three propeptide variants were potent inhibitors of recombinant FhCL1, reducing its activity by ~ 85%, ~ 90% and ~ 95%, respectively. The variant ppFhCL3_Glu^68^/Arg^68^ exhibited low level activity (< 20%) against FhCL2 and FhCL3 but the variants ppFhCL3_Leu^66^/Gly^66^ and ppFhCL3_Tyr^46^Lys^47^/Ala^46^Ala^47^ were not significantly inhibitory (Fig. 7).

### Impact of substituting FhCL3 propeptide key binding residues on potency and selectivity of mature domain binding

The impact of specific amino acid substitutions on the inhibitory activity of ppFhCL3 towards different cathepsin peptidases was assessed in more detail by enzyme kinetics, specifically by determination of the inhibition constants (*K*_*i*_) (Table 2 and Additional file 3). These studies allowed us to make the following observations:

- Consistent with our previous experiment, the substitutions made in the three variants had no or only a relatively small impact on propeptide inhibition of FhCL1 compared to the wild-type. The high potency and selectivity of the wild-type ppFhCL3 towards the parasite cathepsin L peptidases, particularly FhCL3, was demonstrated by their extremely low *K*_*i*_ values; for FhCL1, FhCL2 and FhCL3, these were 0.04 nM (±0.006), 0.004 nM (±0.002) and < 0.002 nM, respectively.
- The *K*_*i*_ value for FhCL3 inhibition was at least 1000-fold lower than the *K*_*i*_ values obtained for human cathepsins, 26.6 nM (±0.4) for HsCL and 2.0 nM (±0.5) for HsCK demonstrating its high specificity.
- Consistent with the results obtained in the enzymatic assays shown in Fig. 7, the activity of double variant ppFhCL3_Tyr^46^Lys^47^/Ala^46^Ala^47^ was dramatically abolished compared to the wild-type propeptide, such that accurate *K*_*i*_ values could not be determined for the parasite cathepsin peptidases FhCL2 and FhCL3 and human peptidases HsCL and HsCK and, thus, were designated as > 10,000 nM.
- The substitution given rise to the variant ppFhCL3_Leu^66^/Gly^66^ had a detrimental effect on propeptide binding to FhCL2, FhCL3, HsCL and HsCK. While this variant showed a significant inhibition of the mature FhCL3 (*K*_*i*_ *=* 8 nM ±2.3), this is ~ 4000-fold lower than the inhibition obtained with the wild-type propeptide. This variant, however, did not alter the propeptide binding to FhCL1.
- The variant ppFhCL3_Glu^68^/Arg^68^ did not have a major impact on the ability of the ppFhCL3 to inhibit the parasite cathepsin L peptidases, which is illustrated by the low *K*_*i*_ calculated, namely 0.3 nM (±0.001) for FhCL1, 0.02 nM (±0.01) for FhCL2 and 0.002 nM (±0.001) for FhCL3.
- Unexpectedly, the substitution made in ppFhCL3_Glu^68^/Arg^68^ resulted in an improved inhibition towards the human cathepsin peptidases, shifting the *K*_*i*_ for HsCL to 6.04 nM (±0.44) and for HsCK to 1.40 nM (±0.2), which indicates that Glu^68^ residue plays an important role in the selectivity of the FhCL3 propeptide (Table 2).

**Table 2:** Inhibition constants calculated for the inhibition of wild-type and variant ppFhCL3 against recombinant *F. hepatica* and human cathepsin peptidases

Cathepsin	Inhibition *K*_*i*_ (nM)			
	ppFhCL3	ppFhCL3 Tyr^46^Lys^47^/Ala^46^Ala^47^	ppFhCL3 Leu^66^/Gly^66^	ppFhCL3 Glu^68^/Arg^68^
FhCL1	0.04 (±0.006)	1.26 (±0.2)	0.05 (±0.004)	0.3 (±0.001)
FhCL2	0.004 (±0.002)	> 10.000*	> 10,000	0.02 (±0.01)
FhCL3	< 0.002*	> 10.000	8.00 (±2.3)	0.002 (±0.001)
HsCL	26.6 (±0.4)	> 10.000	> 10.000	6.04 (±0.44)
HsCK	2.0 (±0.5)	> 10.000	> 10.000	1.40 (±0.2)

* *Ki v*alues > 10,000 nM and < 0.002 could not be calculated accurately

### The synthetic peptide derived from FhCL3 propeptide exhibits low inhibitory activity

**Fig. 8:**
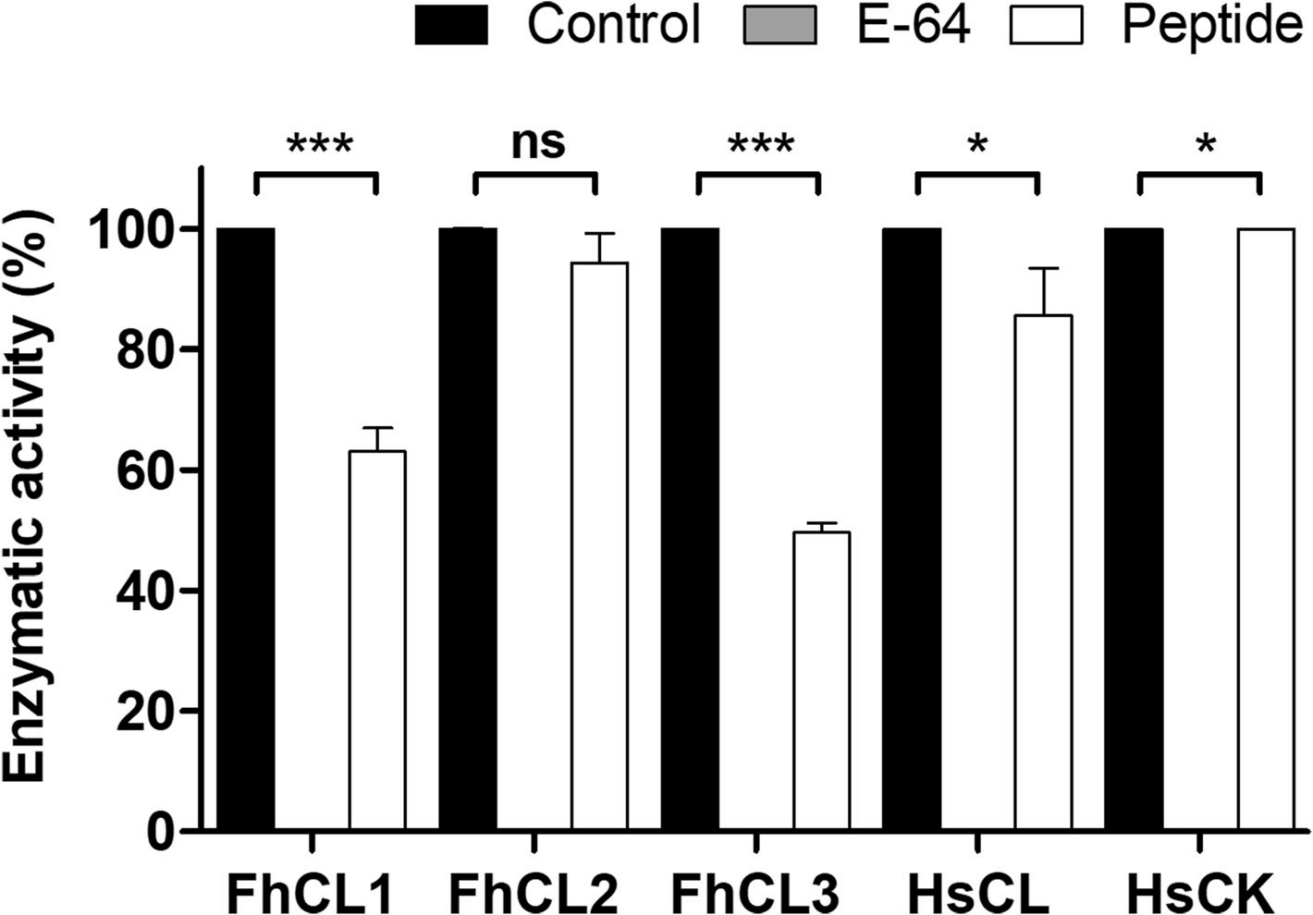
Inhibition profile of the 33-mer synthetic peptide derived from the FhCL3 propeptide. The inhibitory activity of the synthetic peptide at a concentration of 500 nM was tested against the recombinant *F. hepatica* cathepsin peptidases, FhCL1, FhCL2 and FhCL3, and human cathepsin peptidases, HsCL and HsCK. The percentage of inhibition by the peptide is presented relative to the total activity of each enzyme alone. The broad-spectrum cysteine peptidase inhibitor E-64 was used as a positive inhibitor. The experiments were performed in triplicate and the results are presented as mean ± standard deviation. Statistical analysis was carried out using One-way ANOVA with Dunnett multiple comparison (*** *P ≤* 0.001, * *P ≤* 0.05, ns, no-significant differences)

As FhCL3 is highly expressed by the invasive *F. hepatica* NEJs, it is considered a target for the development of drugs and vaccines against *F. hepatica* infections. Impairing FhCL3 activity could prevent invasion, as well as the pathogenesis associated with the parasite invasion and migration through host tissues [23, 24]. Considering the potent inhibitory activity of ppFhCL3 towards parasite cathepsin L peptidases, we designed a 33-mer peptide (p45 to p77) that contained those amino acid residues involved in key interactions with the mature cathepsin domains and also included the conserved motif GNFD (Fig. 5b). The data obtained from the enzymatic assays of the parasite and human cathepsin peptidases in the presence of the peptide, show that the 33-mer peptide did not exhibit potent inhibitory activity. Even at the concentration of 500 nM it reduced the activity of FhCL3 by only ~ 50% and FhCL1 by ~ 30%, and had no significant effect on FhCL2, HsCL and HsCK (Fig. 8).

## Discussion

The cathepsin L cysteine peptidase, FhCL3, plays a critical role in the invasion of the host by *F. hepatica* parasites. Accordingly, the peptidase is highly expressed and secreted by the invasive NEJs that emerge from cysts in the small intestine and rapidly traverse the host intestinal wall. The peptidase is released into the host environment where, along with the parasite’s forward mechanical action, it facilitates penetration of the endothelial layer and underlying lamina propria [7, 10, 11, 25]. Parasites have been observed burrowing through this layer, and even muscular tissue, without hindrance [26] owing to the effectiveness of the parasite’s tissue-penetrating machinery.

Little is known, however, regarding the control of the penetrating process. From a fundamental point of view, it is clear that regulation of the release and activation of FhCL3 is essential to prevent over-destruction of host tissues and to limit the host’s inflammatory responses to tissue damage. Moreover, timely and controlled release of the peptidase is necessary to prevent destruction of the parasite’s own tissues. Like all members of the papain-like cysteine peptidase family, FhCL3 is expressed as an inactive zymogen and requires catalytic removal of its pro-domain or propeptide to become a mature active peptidase [12, 13]. The propeptide is a natural inhibitor of the enzyme and, therefore, functions as a self-regulator of peptidase activity; however, the propeptide must be removed rapidly when required and in the appropriate location. Our studies using a functionally active recombinant FhCL3 demonstrated that the enzyme can activate in vitro at low pH from the 37 kDa zymogen by the autocatalytic release of the ~ 12 kDa propeptide to reveal the 25 kDa mature active domain. These data are consistent with our previous studies on FhCL1 which is produced by adult parasites that reside in the bile ducts [27, 28] and also with studies showing the low pH dependency for activation for human pro-cathepsins L and B [13, 29–32].

An additional or alternative activation process proposed for cathepsin-like peptidases of worms involves trans-activation by an asparaginyl endopeptidase (legumain) that cleave at a specific site between the propeptide and mature enzyme [16, 33]. This mechanism has been shown to occur in the related parasite, *Schistosoma mansoni*, but has yet to be demonstrated in *F. hepatica* [34]*.* Transcriptomic/proteomic studies have recently shown that NEJs express several secreted legumains. Unexpectedly, the most abundant of these enzymes exhibits a Cys to Ser substitution in the active site that would render the enzyme inactive [9, 35]. Thus, the role of legumain peptidases in the activation of FhCL3 and other peptidases in NEJs is still uncertain.

Immunolocalisation studies using polyclonal antibodies directed to the propeptide and to the zymogen suggest that FhCL3 is produced as an inactive zymogen in the gastrodermal cells of the NEJs gut. Immunolabelling with anti-propeptide was significantly weaker than the signal obtained with antibodies against FhCL3 zymogen and was further reduced in NEJs that were cultured for 24 h post-excystment. This may reflect the activation process and secretion of the mature enzyme as we detected potent FhCL3 peptidase activity released into culture medium. Taken together, we propose that the FhCL3 zymogen is secreted into the low-pH lumen of the NEJs gut whereupon it is activated autocatalytically and/or by trans-activation before release into the host tissues.

Interestingly, we have shown that, once activated, the mature FhCL3 exhibits an optimum pH for activity against fluorogenic peptide substrates as well as macromolecules, such as collagen, in the neutral pH range, which is consistent with its role in helping the NEJs transverse the tissues of the intestinal wall [4, 11, 27]. Since studies have shown that a strong immunological response to epitopes present within both propeptide and mature domain is elicited in *F. hepatica*-infected animals it is clear that the parasite also secretes the propeptide into the host tissues [32, 36, 37]. Therefore, the removal of the propeptide in the gut lumen before secretion into the tissues needs to be rapid to prevent re-binding of the released propeptide to the mature FhCL3 peptidase at neutral pH which would impair its activity and delay the parasite’s invasiveness. It may also represent a parasite strategy to avoid a host immune response that could prevent peptidase activation. Indeed, recently Buffoni et al. [38] have shown that immune responses in animals protected by vaccination with FhCL1 were directed to peptides that span the junction between propeptide and the mature domain of the peptidase.

Propeptides have been long known to be potent inhibitors of their cognate enzymes [13, 15]. Nonetheless, little is known about the inhibitory mechanism and selectivity amongst the propeptides of the various members of the *F. hepatica* cathepsins L family. The family, which expanded by gene duplication followed by divergence, contains a number of clades (FhCL1 to 5) that are temporarily expressed in correlation with the migration of the parasite from the intestine to liver and then the bile duct. This switching from one cathepsin L peptidase type to another reflects the differences in their diverged active site composition and hence substrate specificity which, in turn, allows the parasite to confront different tissue macromolecules it encounters. For example, while the migrating immature stages express FhCL3 and FhCL2 that exhibit collagenase activity required for tissue penetration and feeding, the obligate blood-feeding adult parasite produces predominately FhCL1 in the bile duct. The active site changes that occurred in these peptidases would necessitate corresponding alterations within their propeptides to ensure their specific inhibition and control. Thus, we found that the propeptide of FhCL3 exhibited most potent inhibitory activity against this enzyme (*K*_*i*_ < 0.002 nM) compared to FhCL2 (*K*_*i*_ = 0.004 nM) and FhCL1 (*K*_*i*_ = 0.04 nM).

The selectivity by which the ppFhCL3 inhibits its cognate mature enzyme was more apparent when comparing its ability to inhibit the homologous human cathepsin peptidases, HsCL and HsCK; the *K*_*i*_ values for these enzymes were at least 1000-fold less. Despite this, the *K*_*i*_ values of 26.6 nM and 2.0 nM, respectively, show that the ppFhCL3 is still a significant inhibitor of these human enzymes. This reflects overall conservation of the conformation of the ppFhCL3 to the propeptides of HsCL and HsCK. Kwon et al. [21] recently found that a recombinant propeptide of a cathepsin L from the plant *Calatropis procera* potently inhibited HsCL (*K*_*i*_ *=* 2.3 nM) and attributed such inhibition to the high homology between the plant and the human cathepsin zymogens. In particular, all propeptides contain two conserved motifs, ERFNIN and GNFD [13], which are associated with propeptide scaffold stabilization and contain regulatory elements that are important in mediating mature peptidase inhibition [15, 39]. Interestingly, the conservative substitution of DRWNIN found in the ppFhCL3 is also found in the propeptide of HsCK and possibly contributed to the lower *K*_*i*_ values obtained for this enzyme.

To delve more closely into the inhibitory mechanism and selectivity of ppFhCL3 towards different *F. hepatica* and human cathepsins L, a 3-D model of FhCL3 was constructed by homology with FhCL1 [12] and employed in in silico structural analyses. We discovered that interactions made by a pair of residues present in the second loop of the ppFhCL3 stabilizes the propeptide binding with the propeptide binding loop (PBL) within the FhCL3 mature domain; Tyr^46^ forms a hydrogen bond with Asp^228^ and π-π-interaction with Phe^237^, whereas Lys^47^ forms a salt bridge with Glu^233^ and a hydrogen bond with Ser^234^. These interactions were also critical in orientating the C-terminal portion of the propeptide toward the active site which it enters and blocks access to substrates, thus acting as an inhibitor. We found that two significant interactions occur between residues in this portion of the ppFhCL3 and the substrate binding cleft that secure the binding of the propeptide; the Leu^66^ sits in the hydrophobic pocket composed of Ala^255^, Phe^229^, His^249^ and Trp^271^ while the Glu^68^ forms a hydrogen bond with Thr^248^.

We experimentally determined the relevance of these interactions by expressing a series of recombinant variants of ppFhCL3 and testing their inhibitory activity in parallel with the wild-type propeptide. The introduction of alanine residues to abolish ionic interactions in the variant ppFhCL3_Tyr^46^Lys^47^/Ala^46^Ala^47^ resulted in complete lack of activity of this propeptide towards FhCL3 and all other enzymes, reflected in the *K*_*i*_ values > 10.000 nM, and proved the critical importance of these residues in propeptide binding. Likewise, substitution of the large hydrophobic leucine for a small glycine residue in ppFhCL3_Leu^66^/Gly^66^ abrogated propeptide binding. Even when assayed against FhCL3 the *K*_*i*_ value obtained for this propeptide was 8.00 nM ± 2.3, which was 4000-fold higher than the *K*_*i*_ obtained with the wild-type ppFhCL3. Such detrimental effects on binding reflects the inability of the replacement glycine to sit into the hydrophobic pocket of the active site.

Despite our 3-D model indicating the importance of Glu^68^ in propeptide binding to the active site, the propeptide variant that substituted this negatively-charged residue for a positive arginine, ppFhCL3_Glu^68^/Arg^68^, did not demonstrate a major disruption of inhibitory potency towards the *F. hepatica* cathepsin L peptidases. However, we found that this variant propeptide became more selective for HsCL, with *K*_*i*_ values at least 4 times lower than the obtained with the wild-type ppFhCL3. Examination of our in silico models of HsCL indicates that a hydrogen bond between Glu^68^ and aspartic acid (Asp^275^) could be made and has the capacity to improve binding (see Table 1). Thus, the residue at Glu^68^ in the propeptide may not have a major impact on propeptide binding but could influence the specificity due to its freedom to form hydrogen bonds within the active site.

The ability of propeptides to exhibit highly potent inhibition of their cognate enzyme but also cross-inhibit related peptidases suggests that they could be exploited to develop selective and/or broad inhibitors with commercial or bio-therapeutic applications [15, 21]. We attempted to design a small-molecular inhibitor for FhCL3 by synthesising a 33-mer peptide that incorporated the two critical binding regions of the propeptide identified in this study. Despite the 33-mer peptide containing these key amino acid residues, it exhibited weak inhibitory properties that highlighted the importance of overall conformation of the entire 100-residue propeptide. Although the N-terminal portion of the propeptide does not interact directly with the enzyme active site, it stabilises the backbone of the C-terminal part of the propeptide during binding, which is essential for proper enzyme inhibition; truncation of the N-terminal Alpha 1 and 2 helices of human cathepsin L results in over 33,000-fold reduction in the inhibition [13, 32, 40]. Similarly, Korde et al. [41] found that a synthetic 15-mer residue peptide based on the propeptide sequence of falcipain-2, a *Plasmodium falciparum* cathepsin L, inhibited its cognate enzyme with 10,000 times less potency than the propeptide of falcipain-2 itself [41, 42]. Therefore, despite the enthusiasm for exploiting the C-terminal portion of propeptides for small drug design against cathepsin-like peptidases, creating the 3-D configuration that binds the substrate cleft with high affinity will be challenging [13, 18, 22]. More creative design methods such a cyclisation of peptides to improve their conformation, stability and activity compared to linear peptides [43, 44] may be a future option to develop specific propeptide-based inhibitors against the cathepsin peptidases of *F. hepatica* that could have applications as anti-parasitic reagents against this other medically-important parasites.

## Conclusions

Despite the importance of the cathepsin peptidases in parasite virulence, infection and survival, we had very limited knowledge regarding the regulation of their activity. While synthesised as inactive zymogens to prevent unwarranted activation, the mechanisms of inhibition and selectivity by which propeptides control inhibition and activation is unknown. The zymogen of FhCL3, the peptidase critical for the entry of the parasite *F. hepatica* into its host, is synthesised by gastrodermal cells and is activated in the acidic lumen of the gut before being released into the host tissues. We demonstrated that the propeptide of FhCL3 is a highly potent and selective inhibitor of *F. hepatica* and host cathepsin L peptidases, but especially of its cognate mature FhCL3. Our structural, mutagenesis and enzyme kinetics data highlighted the importance of defined molecular interactions between ppFhCL3 and the mature domain enzyme; the residue pair Tyr^46^ and Lys^47^ bind to the PBL, orienting the C-terminal portion of the propeptide towards the enzyme substrate binding cleft, where Leu^66^ penetrates a hydrophobic pocket and Glu^68^ forms hydrogen bonds with an available acceptor within the active site to secure the propeptide in place. Thus, these specific and interdependent propeptide attachment sites act in a “clamp-like” mechanism to prevent substrate access to the active site and maintain the FhCL3 as an inactive zymogen. This clamp-like mechanism is disrupted by low pH that disrupts ionic and hydrogen bonding, particularly at the Tyr^46^/Lys^47^ site. Our data provides interesting molecular and biological insights into the adaptation of parasites to their hosts and new structural information regarding propeptide-cathepsins L inhibition and selectivity. It also offers a starting point for new creative considerations for the design of small-molecule inhibitors that could hinder the activity of FhCL3 and other peptidases and lead to new treatments for the disease caused by *F. hepatica*, and other medically important worm parasites.

## Methods

### Heterologous expression of the recombinant *F. hepatica* cathepsin L3 propeptide

The FhCL3 propeptide (ppFhCL3) sequence was designed based on the full gene sequence of the *F. hepatica* cathepsin L3 zymogen (MT876407) used for recombinant expression of the FhCL3 zymogen (Fig. 5).

The FhCL3 propeptide (ppFhCL3) sequence was codon optimised for expression in *E. coli* cells and synthesised in the pET-28a(+) vector (kanamycin resistant) with a C-terminal His-tag (Genscript). The synthesised vector was transformed into BL21 competent *E. coli* cells (ThermoFisher Scientific), which were cultured in LB broth containing kanamycin (1 μg/mL) at 37 °C. Once an OD600 was reached, protein expression was induced with 1 mM isopropyl-β-D-1-thiogalactopyranoside (IPTG; ThermoFisher Scientific) for 3 h at 30 °C. Following centrifugation at 10,000 x *g* for 10 min at 4 °C, the bacteria pellet was digested with lysozyme (10 μg/mL) and sonicated. Subsequent centrifugation at 10,000 x *g* for 10 min at 4 °C was used to recover the soluble recombinant ppFhCL3 within the supernatant that was purified using the Profinia Affinity Chromatography Protein Purification System (Bio-Rad), with the mini profinity IMAC and mini Bio-Gel P^− 6^ desalting cartridges (Bio-Rad). The protein concentration and purity were verified by Bradford Protein Assay (Bio-Rad) and by 4–20% SDS-PAGE gels (Bio-Rad) stained with Biosafe Coomassie (Bio-Rad), respectively. The gels were visualised using a G:BOX Chemi XRQ imager (Syngene).

### *F. hepatica* NEJs and adult parasites and extracts

*F. hepatica* metacercariae, Italian isolate, were purchased from Ridgeway Research (UK). The newly excysted juveniles (NEJs) were obtained by excysting the metacercariae as previously described by Robinson et al. (2009). Briefly, after removing the outer cyst wall, the metacercariae were incubated in excystment medium (1.2% sodium bicarbonate, 0.9% sodium chloride, 0.2% sodium tauroglycocholate, 0.006% L-cysteine [w/v] in a 0.07% hydrochloric acid solution [v/v]) for up to 3 h at 37 °C, in 5% CO_2_. NEJs were subsequently washed and transferred into RPMI-1640 (ThermoFisher Scientific), pH 7.3, using a Gilson pipette. To produce somatic extracts from NEJs the parasites were freeze-thawed and homogenised using a sterile pestle to extract the proteins. Following centrifugation at 10,000 x *g* for 40 min at 4 °C, the somatic proteins within the supernatant were collected and the protein concentration measured by the Bradford Protein Assay (Bio-Rad).

Adult flukes were obtained from the livers of sheep orally infected with 120 *F. hepatica* metacercariae (Italian isolate: Ridgeway Research Ltd) administered in water, carried out at Agri-Food and Biosciences Institute (AFBI) under license from the Department of Health, Social Services and Public by the Animal (Scientific Procedures) Act 1986 (License No. PPL 2801), after ethical review by the AFBI Animal Ethics Committee. ES products from *F. hepatica* adult worms was prepared as previously described by Cwiklinski et al. [45]. Briefly, the recovered flukes were washed in 1x PBS to eliminate contamination from their intestinal contents. Then, the parasites were cultured (2 worms/mL) in RPMI 1640 medium supplemented with 0.1% [w/v] glucose, 100 U penicillin and 100 mg/mL streptomycin (Sigma-Aldrich) for 5 h at 37 °C. The culture supernatant was collected, centrifuged at 700 x *g* for 30 min to remove large debris and then concentrated using Amicon Ultra 3 kDa columns (Merck Millipore). Protein concentration of all ES and somatic extracts were verified by Bradford Protein Assay (Bio-Rad) and adjusted to ~ 1 mg/mL with PBS.

### Immunolocalisation of FhCL3 zymogen and propeptide within *F. hepatica* NEJs by confocal microscopy

Polyclonal antibodies against recombinant ppFhCL3 and recombinant FhCL3 zymogen were obtained from rabbits immunised with each of the respective recombinant proteins (Eurogentec). *F. hepatica* NEJs 6 h and 24 h post-excystment were fixed in 4% paraformaldehyde (Sigma-Aldrich) for 1 h at room temperature (RT). After three washes with antibody diluent (AbD: 0.1% [v/v] Triton X-100, 0.1% bovine serum albumin and 0.1% [w/v] sodium azide in 1x PBS), the NEJs were probed with either anti-ppFhCL3, anti-FhCL3 or rabbit pre-immune antiserum diluted 1:500 in AbD buffer, overnight (ON) at 4 °C. After three washes in AbD, the secondary antibody, fluorescein isothiocyanate (FITC)-labelled goat anti-rabbit IgG (Sigma-Aldrich) (1:200), was added and incubated ON at 4 °C. Phalloidin-tetramethylrhodamine isothiocyanate (TRITC) (200 μg/mL [w/v]) (Sigma-Aldrich) was used to counter-stain the muscle tissue and provide structure. All the specimens were whole-mounted in a glycerol solution containing 0.1 M propyl gallate and visualised in a confocal scanning laser microscopy (CSLM) (Leica TCS SP5) under the HCX PL APO CS 100x oil objective lens, using a Leica type F immersion oil.

### Immunodetection of native FhCL3 zymogen and propeptide in *F. hepatica* NEJs protein extract

The recombinant proteins, FhCL3 zymogen (1 μg/lane) and ppFhCL3 (1 μg/lane), and the somatic extracts (15 μg/lane) of NEJs were resolved in 4–20% SDS-PAGE gels (Bio-Rad) and electro-transferred onto nitrocellulose membranes. The membranes were incubated in blocking solution (5% [w/v] milk, 0.05% [v/v] Tween-20 in 1x PBS) for 1 h at RT, and then probed with the primary antibodies anti-ppFhCL3, anti-FhCL3 or rabbit pre-immune antiserum (1:7500) for 1 h at RT. After five washes in PBST (0.05% [v/v] Tween in 1x PBS), the secondary antibody, an alkaline phosphatase-conjugated goat anti-rabbit IgG (Sigma-Aldrich) (1:5000), was added to the membranes, which were incubated for 1 h at RT. The Western blots were developed with the chromogenic substrate SIGMA FAST BCIP/NBT (5-Bromo-4-chloro-3-indolyl phosphate/Nitro blue tetrazolium) (Sigma-Aldrich) and imaged using a G:BOX Chemi XRQ imager (Syngene).

### Pull-down of *F. hepatica* adult excreted-secreted proteins by the recombinant FhCL3 propeptide

The recombinant ppFhCL3 (1 μM), was added to 10 μL bed volume of pre-washed Ni-NTA beads (Qiagen) and incubated with agitation for 1 h at RT. The beads were washed three times with RPMI-1640 media, added to the ES products from *F. hepatica* adult worms (40 μg) and incubated with agitation for another 1 h at RT. After three washes with washing buffer (50 mM NaH_2_PO_4_, 300 mM NaCl, 10 mM C_3_H_4_N_2_), elution buffer (50 mM NaH_2_PO_4_, 300 mM NaCl, 250 mM C_3_H_4_N_2_) was added to the beads and incubated with agitation for 10 min at RT. The eluted proteins were recovered in the supernatant by centrifugation at 400 x g for 5 min and verified in a 4–20% SDS-PAGE gel (Bio-Rad) under reducing conditions, stained with Biosafe Coomassie (Bio-Rad). After the gels were imaged using a G:BOX Chemi XRQ imager (Syngene), the band observed at ~ 25 kDa was carefully excised from the gel and analysed by liquid chromatography–mass spectrometry (LC-MS/MS) at the Fingerprints Proteomics Facility, University of Dundee, Scotland.

### Molecular modelling of the *F. hepatica* FhCL3 propeptide interaction with the FhCL3 mature enzyme

The FhCL3 homology model was built based on the crystal structure of FhCL1 previously reported by us (PDB code: 2O6X) [12] (sequence identity: 71%), using the Prime program of the Schrodinger software [46]. The ppFhCL3 homology model was built using the prosegment of the human pro-cathepsin L (PDB code: 1CJL) (sequence identity is 30%) by the Prime module. The initial coordinates of FhCL3-ppFhCL3 complex were obtained by superimposition with the complex of the human pro-cathepsin L (PDB code: 1CJL). The sequence identity between FhCL3 and human cathepsin L is 48%. Next, the FhCL3-ppFhCL3 complex was subjected to minimisation and molecular dynamic simulations the MacroModel module protocols of the Schrodinger software [46]. Default protocols with 5000 steps of minimisation and 5 ns of molecular dynamics simulations in implicit solvent at temperature 300 K were used to obtain the final complex. The OPLS_2005 force field was used in MacroModel calculations. The image with the molecular models was prepared with the Maestro program of Schrodinger software [46]. This analysis together with sequence alignment allowed the identification of key-residues participating in the binding and stabilisation of the ppFhCL3 with FhCL3 and narrowed the propeptide portion that possibly determines the blockage of the active site of the mature cathepsin (see Additional file 4).

### Production of the recombinant FhCL3 propeptide variants and of the synthetic peptide

Three variants of the ppFhCL3 sequence were recombinantly produced in *E. coli* BL21 cells following the same protocol described above. These variants consisted of (a) ppFhCL3_Tyr^46^Lys^47^/Ala^46^Ala^47^, whereby the tyrosine (Tyr) residue at position p46 and a lysine (Lys) at position p47 were substituted by alanine (Ala) residues, (b) ppFhCL3_Leu^66^/Gly^66^, whereby the large hydrophobic leucine (Leu) at position p66 was substituted by a small non-charged glycine (Gly) residue, and (c) ppFhCL3_Glu^68^/Arg^68^, whereby the glutamic acid (Glu) at position p68 was substituted by an arginine (Arg) residue. The portion of the ppFhCL3 predicted to specifically interact and block the active site of the mature cathepsin comprised of 33 amino acids in length (p45 to p77) was chemically synthesised (GL Biochem, Shanghai).

### Inhibition properties of the recombinant FhCL3 propeptide

Unless otherwise stated, all enzymes were assayed in a 200 μL reaction volume using the sodium acetate reaction buffer (100 mM sodium acetate, 1 mM EDTA, 1 mM DTT, 0.01% [w/v] brij L23, pH 7.0). Enzymatic concentration and substrates used in the screening assays are presented in Table 3. Initially, the reaction buffer was mixed with the propeptide inhibitor and the peptidase target was then added to the reaction and incubated for 10 min at 37 °C before the fluorogenic substrate was added. The broad-spectrum inhibitor E-64 (100 μM; Sigma-Aldrich) was used as a positive control inhibitor of cysteine peptidases. Hydrolytic activity was measured over 1 h, at 37 °C as relative fluorescent units (RFU) in a PolarStar Omega Spectrophotometer (BMG LabTech). All assays were carried out in triplicate.

**Table 3:** Assay conditions of each cysteine peptidase screened against the wild-type ppFhCL3, ppFhCL3 variants and synthetic peptide

Enzyme	Substrate
*F. hepatica* cathepsin L1	Z-Leu-Arg-NHMec (20 μM)
(FhCL1; 2.7 nM)	
*F. hepatica* cathepsin L2	Z-Leu-Arg-NHMec (20 μM)
(FhCL2; 5 nM)	
*F. hepatica* cathepsin L3	Z-Gly-Pro-Arg-NHMec (20 μM)
(FhCL3; 5 nM)	
Human cathepsin L	Z-Phe-Arg-NHMec (20 μM)
(HsCL; 0.2 nM)	
Human cathepsin K	Z-Phe-Arg-NHMec (20 μM)
(HsCK; 2 nM)	

#### ppFhCL3 inhibitory activity against native cysteine peptidases in the ES products from *F. hepatica* adult worms

The cysteine peptidase activity of excretory-secretory (ES) products from *F. hepatica* adult parasites was assayed in the presence or absence of ppFhCL3 (10 nM) or the inhibitor E-64. The ES products, 10 μL/well, were initially mixed with the reaction buffer and the substrate Z-Leu-Arg-NHMec (Bachem) (20 μM) was then added to the reaction. Inhibitory activity was calculated relative to the enzymatic activity detected for the ES products alone.

#### FhCL3 propeptide inhibitory activity against recombinant *F. hepatica* and human cathepsin peptidases

The recombinant *F. hepatica* cathepsin L1 (FhCL1), L2 (FhCL2) and L3 (FhCL3) zymogens were produced in yeast and purified in our laboratory, as previously described [10]. For the enzymatic assays, the recombinant *F. hepatica* zymogens were induced to autocatalytically activate by mixing each of them with activation buffer (0.1 M sodium citrate buffer, 2 mM DTT, 2.5 mM EDTA, pH 4.5) and incubating for 2 h (FhCL1 and FhCL2) or 5 h (FhCL3) at 37 °C. The human cathepsin L and K, HsCL and HsCK, were acquired from Sigma-Aldrich and Enzo Life Sciences, respectively.

The inhibitory effect of ppFhCL3 (10 nM) was verified against recombinant *F. hepatica* cathepsin peptidases, FhCL1, FhCL2 and FhCL3, as well as against two human cathepsin peptidases HsCL and HsCK. The effect of pH on the inhibitory activity of the propeptide was verified by performing the same reaction in sodium acetate buffer at pH 4.5, 5.5, 6.0 and 7.0. The percentage inhibition was calculated relative to the activity of the respective enzyme alone, for each pH.

#### The inhibitory activity of FhCL3 propeptide and its variants against native cysteine peptidases excreted-secreted by NEJs

The cysteine peptidases activity in NEJs 24 h excretory-secretory (ES) products were assayed in the presence or absence of 10 nM ppFhCL3, ppFhCL3_Tyr^46^Lys^47^/Ala^46^Ala^47^, ppFhCL3_Leu^66^/Gly^66^, ppFhCL3_Glu^68^/Arg^68^ or E-64. The ES products (10 μL/well) were initially mixed with the reaction buffer and the fluorogenic peptide substrate Z-Gly-Pro-Arg-NHMec (Bachem) (20 μM) was then added to the reaction. Inhibitory activity was calculated relative to the enzymatic activity detected for the ES products alone.

#### The inhibitory activity of FhCL3 propeptide and its variants against recombinant *F. hepatica* and human cathepsin peptidases

The inhibitory specificity of 10 nM wild-type ppFhCL3, ppFhCL3_Tyr^46^Lys^47^/Ala^46^Ala^47^, ppFhCL3_Leu^66^/Gly^66^, ppFhCL3_Glu^68^/Arg^68^ were screened against *F. hepatica* cathepsin peptidases, FhCL1, FhCL2, FhCL3, and the human cathepsin peptidases, HsCL and HsCK. The inhibitory activity was calculated relative to the activity observed for the respective enzymes alone.

The inhibition constants (*K*_*i*_) of the wild-type and variant recombinant FhCL3 propeptides, namely wild-type ppFhCL3, ppFhCL3_Tyr^46^Lys^47^/Ala^46^Ala^47^, ppFhCL3_Leu^66^/Gly^66^, ppFhCL3_Glu^68^/Arg^68^, against the *F. hepatica* or human cathepsin peptidases were determined by decreasing the concentration (nM) of the recombinant propeptide by serial dilution in assays performed under the same conditions as described above. The assay parameters were as follows: FhCL1 activity (0.5 nM) was assayed with the fluorogenic substrate Z-Leu-Arg-NHMec (1.09 μM); FhCL2 activity (1 nM) with the substrate Z-Leu-Arg-NHMec (2.1 μM); FhCL3 activity (5 nM) with the Z-Gly-Pro-Arg-NHMec (10.6 nM); HsCL activity (0.7 nM) was assayed using Z-Phe-Arg-NHMec (2.2 μM); HsCK (2 nM) was assayed using Z-Phe-Arg-NHMec (48.5 μM). The *K*_i_ values were estimated using non-linear regression analysis in GraphPad Prism 5.0 Software (http://www.graphpad.com). Initial velocities were fitted to Morrison’s equation (Eq. 1) and the resulting apparent *K*_i_ (*K*_i_ app) fitted to Eq. 2 to determine the *K*_i_ of the peptidase in the presence of the inhibitor [47]. All the assays were carried out in triplicates.

1 $$ \frac{vi}{vo}=1-\frac{{\left(\left[E\right]+\left[I\right]+{Ki}^{app}-\sqrt{\Big(\left[E\right]+\left[I\right]+{ki}^{app}}\right)}^2-4\left[E\right]\left[I\right]\ }{2\left[E\right]} $$

2 $$ {Ki}^{app}= Ki\ \left(1+\frac{Km}{\left[S\right]}\right) $$

#### The inhibitory activity of the synthetic peptide derived from FhCL3 propeptide against recombinant *F. hepatica* and human cathepsin peptidases

The 33-mer synthetic peptide derived from ppFhCL3 was re-suspended in 1% [v/v] dimethylsulphoxide (DMSO) in sterile water to 1 mg/mL. To test the inhibitory activity of the peptide against the parasite and human cathepsins (FhCL1, FhCL2, FhCL3, HsCL and HsCK) the peptide was diluted to 500 nM in sodium acetate buffer, pH 7.0, and incubated for 1 h at 37 °C. Subsequently, the peptidase was added to the reaction and the assay performed as described above. A blank containing DMSO alone was used as control.

## Supplementary Information

**Additional file 1: Fig. S1.** Inhibition profile of FhCL3 propeptide against recombinant parasite and human cysteine and serine peptidases.

**Additional file 2: Fig. S2.** Recombinant expression of the variant FhCL3 propeptides.

**Additional file 3: Fig. S3A-E.** Inhibition constant of the FhCL3 propeptide and its variants against *F. hepatica* and human cathepsin peptidases.

**Additional file 4: Fig. S4.** Alignment of the *F. hepatica* and human cathepsin peptidases.

**Additional file 5: Fig. S5A-B.** Uncropped SDS-PAGE gels and Western blot images included in the manuscript.

**Additional file 6: Fig. S6A-B.** Antibodies against FhCL3 zymogen differentially recognize the recombinant FhCL3 zymogen, mature domain and propeptide segment.

## Data Availability

The datasets used and/or analysed during the current study available from the corresponding author on reasonable request.

## Abbreviations

Ala: Alanine
Arg: Arginine
Asp: Aspartic acid
Chy: Chymotrypsin
Cys: Cysteine
ES: Excretory-secretory products
FhCB1: *F. hepatica* cathepsin B1 peptidase
FhCB2: *F. hepatica* cathepsin B2 peptidase
FhCB3: *F. hepatica* cathepsin B3 peptidase
FhCL1: *F. hepatica* cathepsin L1 peptidase
FhCL2: *F. hepatica* cathepsin L2 peptidase
FhCL3: *F. hepatica* cathepsin L3 peptidase
FhCL5: *F. hepatica* cathepsin L5 peptidase
Phe: Phenylalanine
FITC: Fluorescein isothiocyanate
Glu: Glutamic acid
Gly: Glycine
His: Histidine
HsCL: Human cathepsin L
HsCK: Human cathepsin K
Kal: Kallikrein
*K*_*i*_: Inhibition constant
LC-MS/MS: Liquid chromatography–mass spectrometry
Leu: Leucine
Lys: Lysine
NEJs: Newly excysted juveniles
PBL: Propeptide binding loop
ppFhCL3: Propeptide of FhCL3
Ser: Serine
TRITC: Phalloidin-tetramethylrhodamine isothiocyanate
Thr: Threonine
Trp: Tryptophan
Try: Trypsin
Tyr: Tyrosine
