# Supplementary material for: Regulation of the Fasciola hepatica newly excysted juvenile cathepsin L3 (FhCL3) by its propeptide: a proposed ‘clamp-like’ mechanism of binding and inhibition
Source: BMC Mol Cell Biol. 2020 Dec 7;21:90. doi: 10.1186/s12860-020-00335-5 (PMC7720491; doi:10.1186/s12860-020-00335-5)
Supplement: Supplementary file 1 — Additional file 1: Fig. S1. Inhibition profile of FhCL3 propeptide against recombinant parasite and human cysteine and serine peptidases. [file 12860_2020_335_MOESM1_ESM.docx]

**Additional file 1**


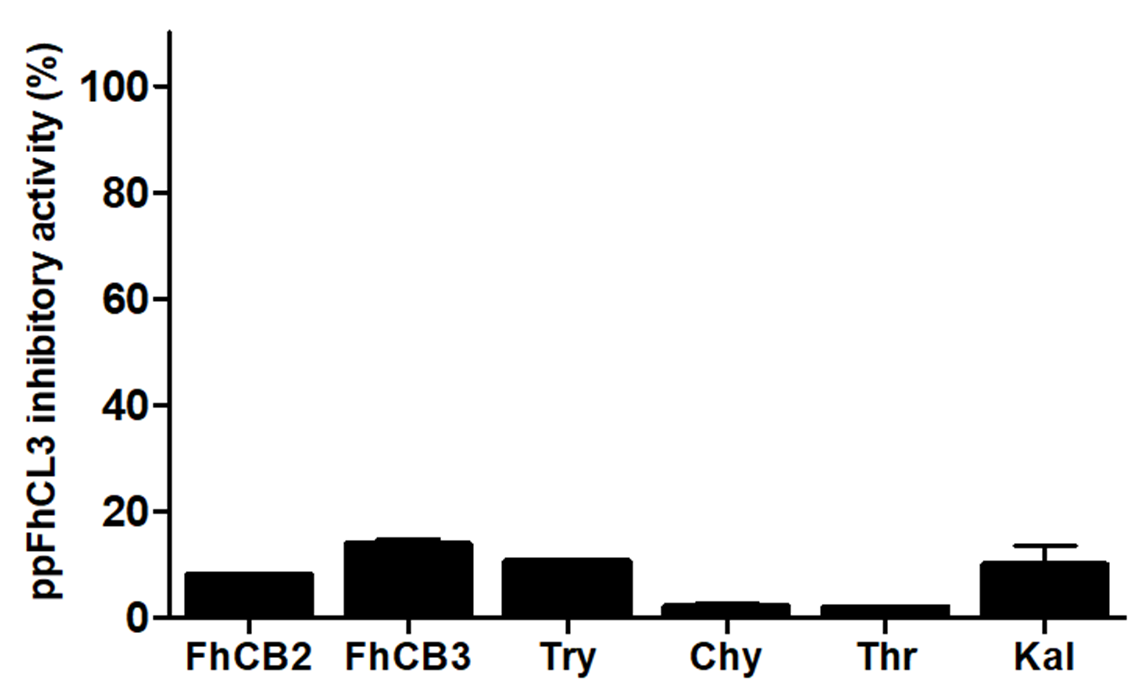


**Additional file 1. Fig. S1. Inhibition profile of FhCL3 propeptide against recombinant parasite and human cysteine and serine peptidases.** The inhibitory activity of ppFhCL3 was examined at 10 nM against recombinant *F. hepatica* cathepsin B peptidases (FhCB2, 200 nM and FhCB3, 100 nM) and against recombinant human serine peptidases trypsin (Try, 168 nM), chymotrypsin (Chy, 4 nM), thrombin (Thr, 800 pM) and kallikrein (Kal, 150 nM). The inhibitory activities are presented relative to the total activity of each enzyme alone. The experiments were performed in triplicate and the results are presented as mean ± standard deviation.
