# Supplementary material for: Regulation of the Fasciola hepatica newly excysted juvenile cathepsin L3 (FhCL3) by its propeptide: a proposed ‘clamp-like’ mechanism of binding and inhibition
Source: BMC Mol Cell Biol. 2020 Dec 7;21:90. doi: 10.1186/s12860-020-00335-5 (PMC7720491; doi:10.1186/s12860-020-00335-5)
Supplement: Supplementary file 2 — Additional file 2: Fig. S2. Recombinant expression of the variant FhCL3 propeptides. [file 12860_2020_335_MOESM2_ESM.docx]

**Additional file 2**


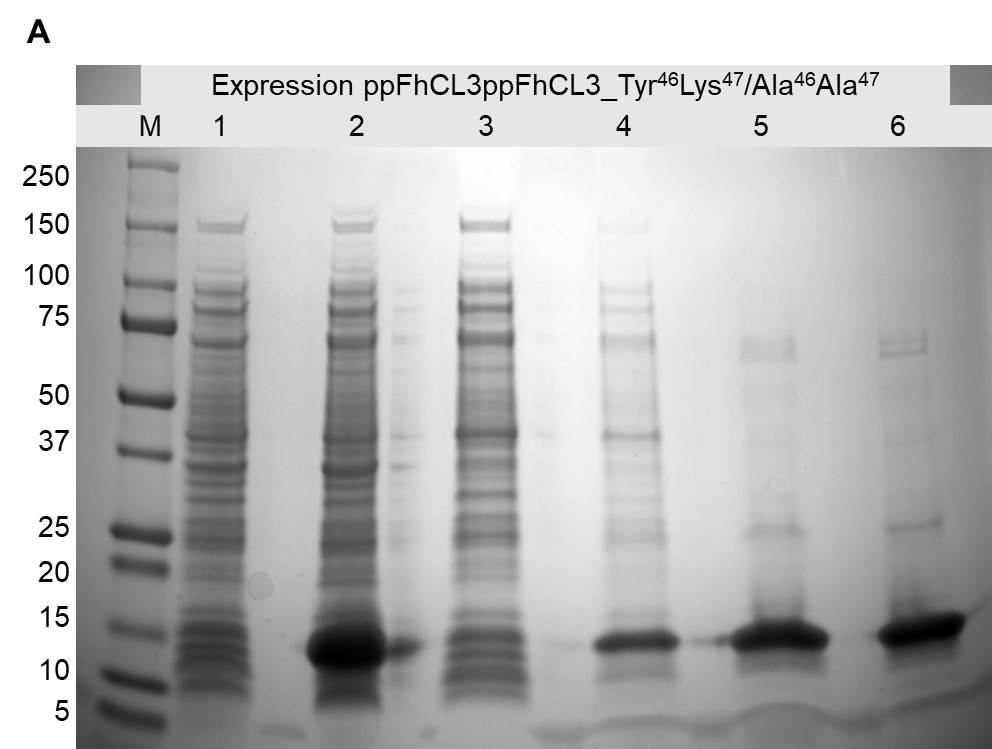


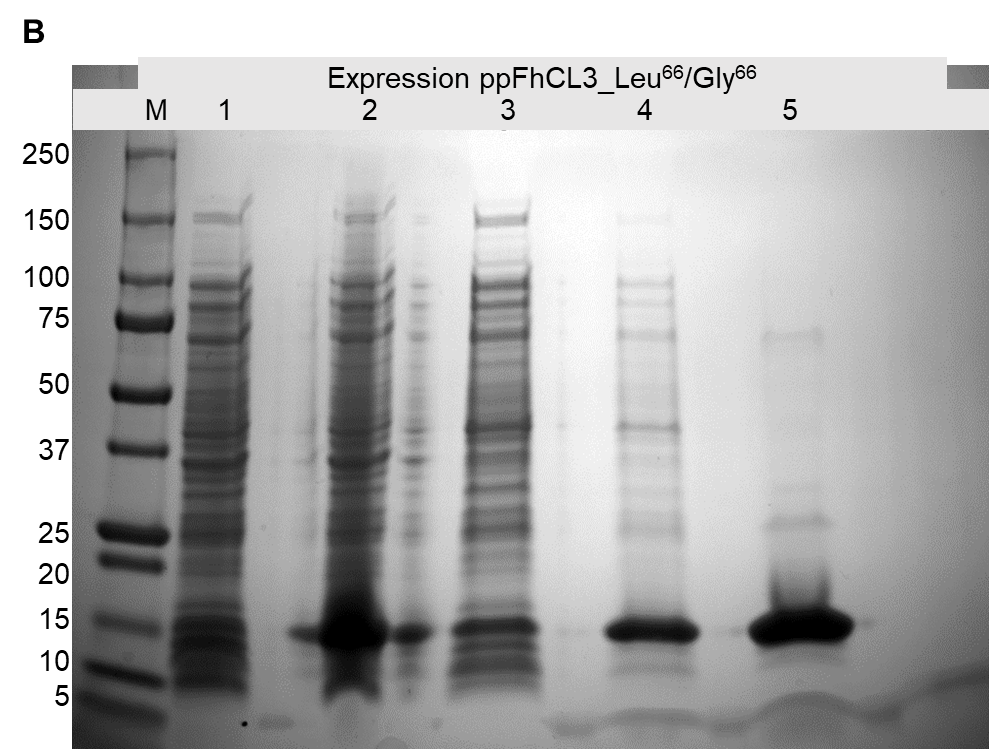


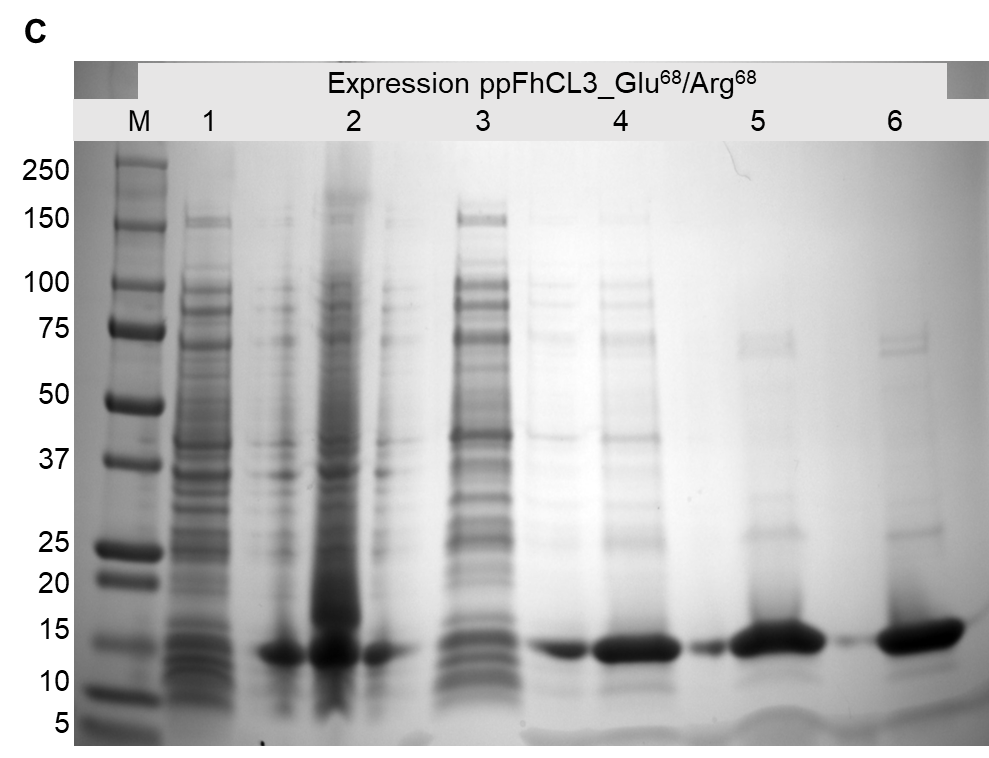


**Additional file 2. Fig. S2. Recombinant expression of the variant FhCL3 propeptides.** The three propeptide variants, ppFhCL3_Tyr^46^Lys^47^/Ala^46^Ala^47^ (A), ppFhCL3_Leu^66^/Gly^66^ (B) and ppFhCL3_Glu^68^/Arg^68^ (C), were recombinantly produced in *E. coli* BL21 cells, purified by affinity chromatography and resolved in 4-12% SDS-PAGE gels to verify their expression and purity. Lane 1, *E. coli* BL21 cell pellet before induction with IPTG; lane 2, cell pellet after induction for 3 hr at 30°C; lane 3, supernatant of the cell pellet after extraction and binding to the affinity column Profinia affinity chromatography system); lane 4, wash after recombinant protein binding to the affinity column; lane 5, the eluted recombinant propeptide variant; lane 6, the dialysed recombinant propeptide variant. M: Molecular weight in kDa.
