# Supplementary material for: Regulation of the Fasciola hepatica newly excysted juvenile cathepsin L3 (FhCL3) by its propeptide: a proposed ‘clamp-like’ mechanism of binding and inhibition
Source: BMC Mol Cell Biol. 2020 Dec 7;21:90. doi: 10.1186/s12860-020-00335-5 (PMC7720491; doi:10.1186/s12860-020-00335-5)
Supplement: Supplementary file 3 — Additional file 3: Fig. S3A-E. Inhibition constant of the FhCL3 propeptide and its variants against F. hepatica and human cathepsin peptidases. [file 12860_2020_335_MOESM3_ESM.docx]

**Additional file 3**


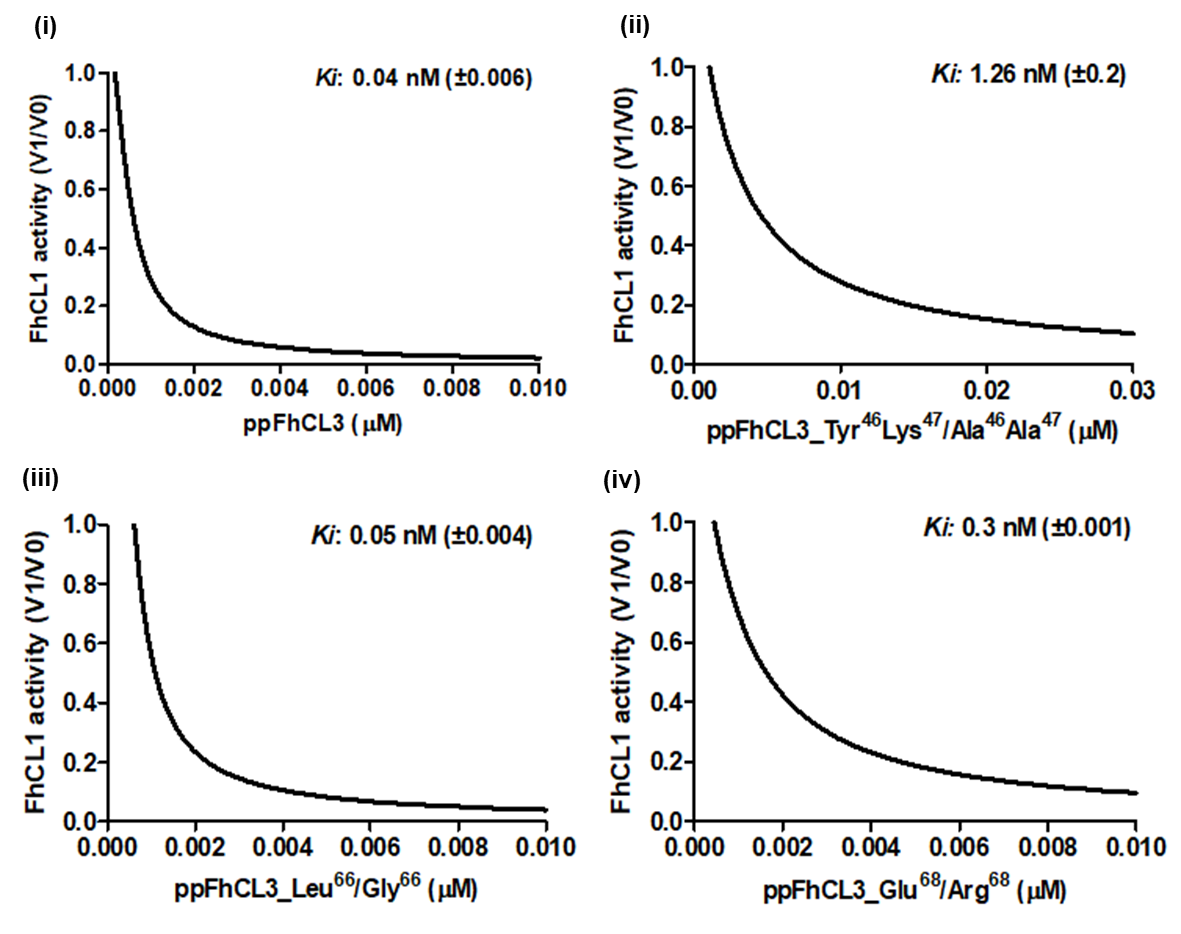


**Additional file 3. Fig. S3A. Inhibition constant of the FhCL3 propeptide and its variants against *F. hepatica* L1.** The FhCL1 (0.5 nM) was assayed in the presence of decreasing concentrations of the (i) ppFhCL3 and its variants (ii) ppFhCL3_Tyr^46^Lys^47^/Ala^46^Ala^47^, (iii) ppFhCL3_Leu^66^/Gly^66^, and (iv) ppFhCL3_Glu^68^/Arg^68^. Inhibition constant (*K_i_*) values are presented as mean ± standard deviation.


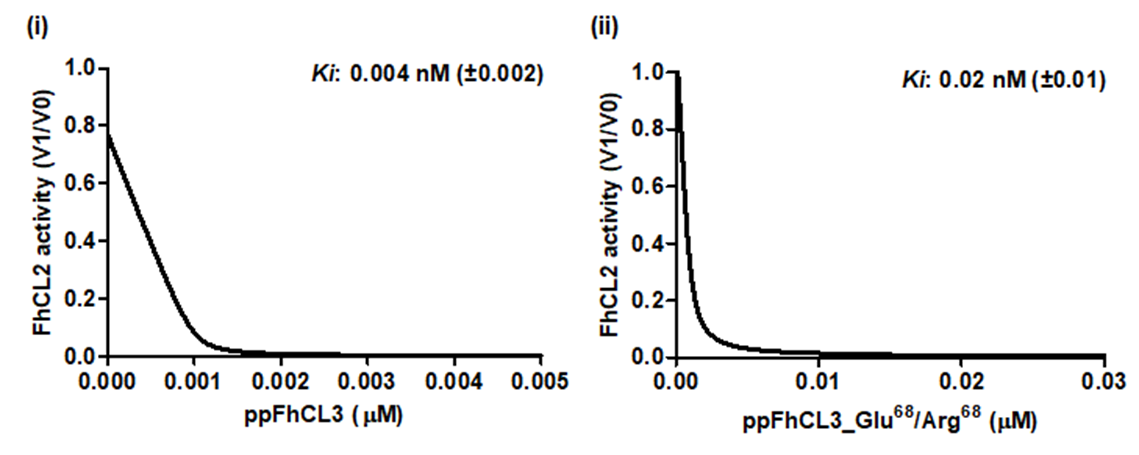


**Additional file 3. Fig. S3B. Inhibition constant of the FhCL3 propeptide and its variants against *F. hepatica* L2.** The FhCL2 (2 nM) was assayed in the presence of decreasing concentrations of the (i) ppFhCL3 and its variant (ii) ppFhCL3_Glu^68^/Arg^68^. Inhibition constant (*K_i_*) values are presented as mean ± standard deviation.

**
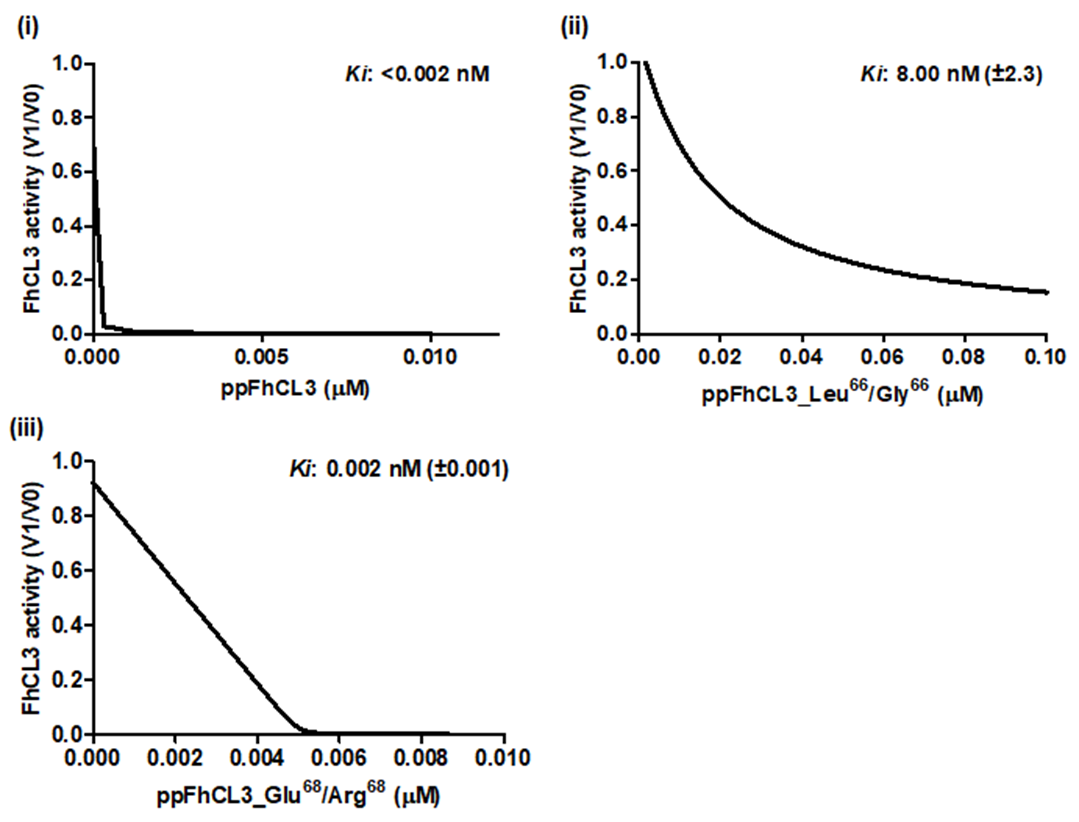
**

**Additional file 3. Fig. S3C. Inhibition constant of the FhCL3 propeptide and its variants against *F. hepatica* L3.** The FhCL3 (5 nM) was assayed in the presence of decreasing concentrations of the (i) ppFhCL3 and its variants (ii) ppFhCL3_Leu^66^/Gly^66^ and (iii) ppFhCL3_Glu^68^/Arg^68^. Inhibition constant (*K_i_*) values are presented as mean ± standard deviation.

**
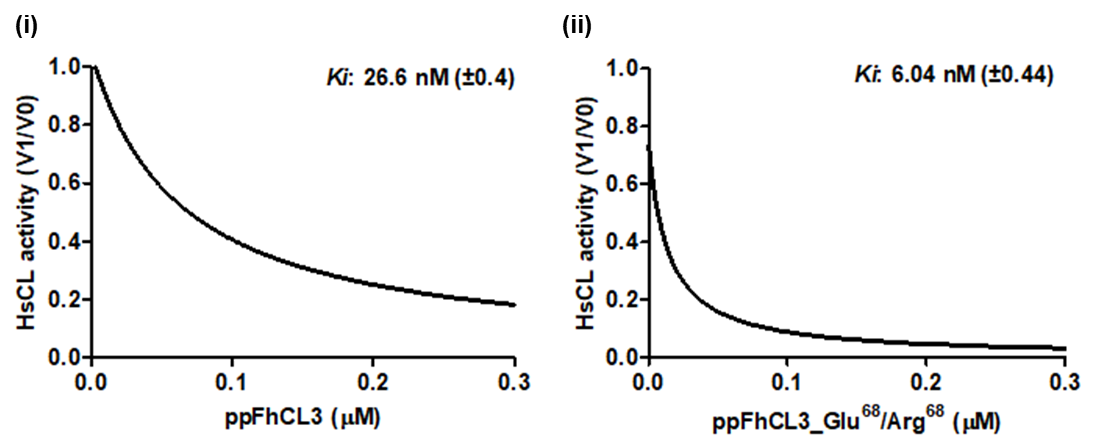
**

**Additional file 3. Fig. S3D. Inhibition constant of the FhCL3 propeptide and its variants against human cathepsin L.** The HsCL (0.7 nM) was assayed in the presence of decreasing concentrations of the (i) ppFhCL3 and its variant (ii) ppFhCL3_Glu^68^/Arg^68^. Inhibition constant (*K_i_*) values are presented as mean ± standard deviation.


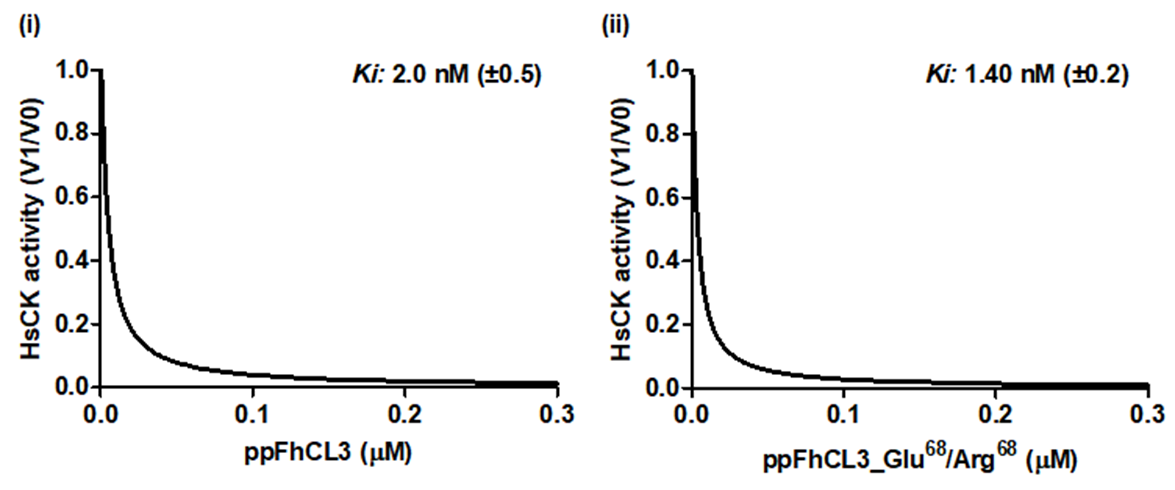


**Additional file 3. Fig. S3E. Inhibition constant of the FhCL3 propeptide and its variants against human cathepsin K.** The HsCK (2 nM) was assayed in the presence of decreasing concentrations of the (i) ppFhCL3 and its variant (ii) ppFhCL3_Glu^68^/Arg^68^. Inhibition constant (*K_i_*) values are presented as mean ± standard deviation.
