# Supplementary material for: Regulation of the Fasciola hepatica newly excysted juvenile cathepsin L3 (FhCL3) by its propeptide: a proposed ‘clamp-like’ mechanism of binding and inhibition
Source: BMC Mol Cell Biol. 2020 Dec 7;21:90. doi: 10.1186/s12860-020-00335-5 (PMC7720491; doi:10.1186/s12860-020-00335-5)
Supplement: Supplementary file 4 — Additional file 4: Fig. S4. Alignment of the F. hepatica and human cathepsin peptidases. [file 12860_2020_335_MOESM4_ESM.docx]

**Additional file 4**


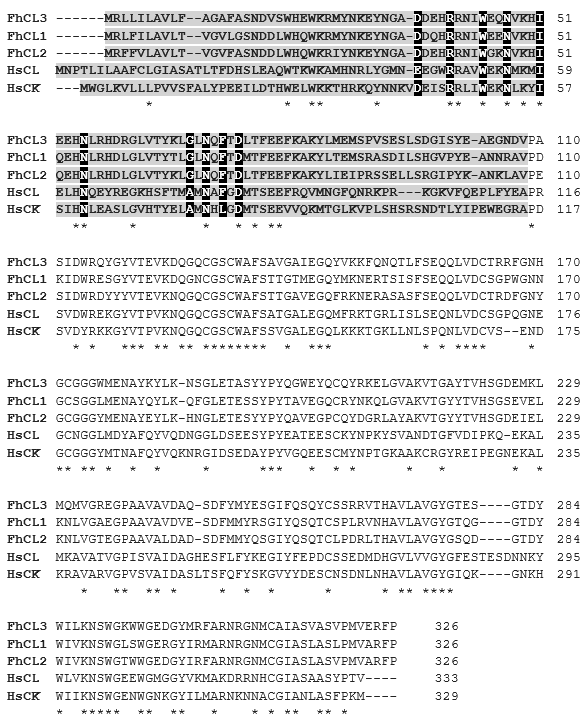


**Additional file 4. Fig S4. Alignment of the *F. hepatica* and human cathepsin peptidases.** Clustal Omega alignment of the *F. hepatica* FhCL3, FhCL2 and FhCL1, and the *Homo sapiens* cathepsin L (HsCL) and cathepsin K (HsCK) amino acid sequences. The propeptide domain of each cathepsin peptidase is shaded in grey and the residues forming the propeptide conserved motifs ERFNIN and GNFD are highlighted in black. Asterisks below the alignment indicate residues identical within sequences.
