# Supplementary material for: Regulation of the Fasciola hepatica newly excysted juvenile cathepsin L3 (FhCL3) by its propeptide: a proposed ‘clamp-like’ mechanism of binding and inhibition
Source: BMC Mol Cell Biol. 2020 Dec 7;21:90. doi: 10.1186/s12860-020-00335-5 (PMC7720491; doi:10.1186/s12860-020-00335-5)
Supplement: Supplementary file 5 — Additional file 5: Fig. S5A-B. Uncropped SDS-PAGE gels and Western blot images included in the manuscript. [file 12860_2020_335_MOESM5_ESM.docx]

**Additional file 5. Uncropped SDS-PAGE gels and Western blot images included in the manuscript.**


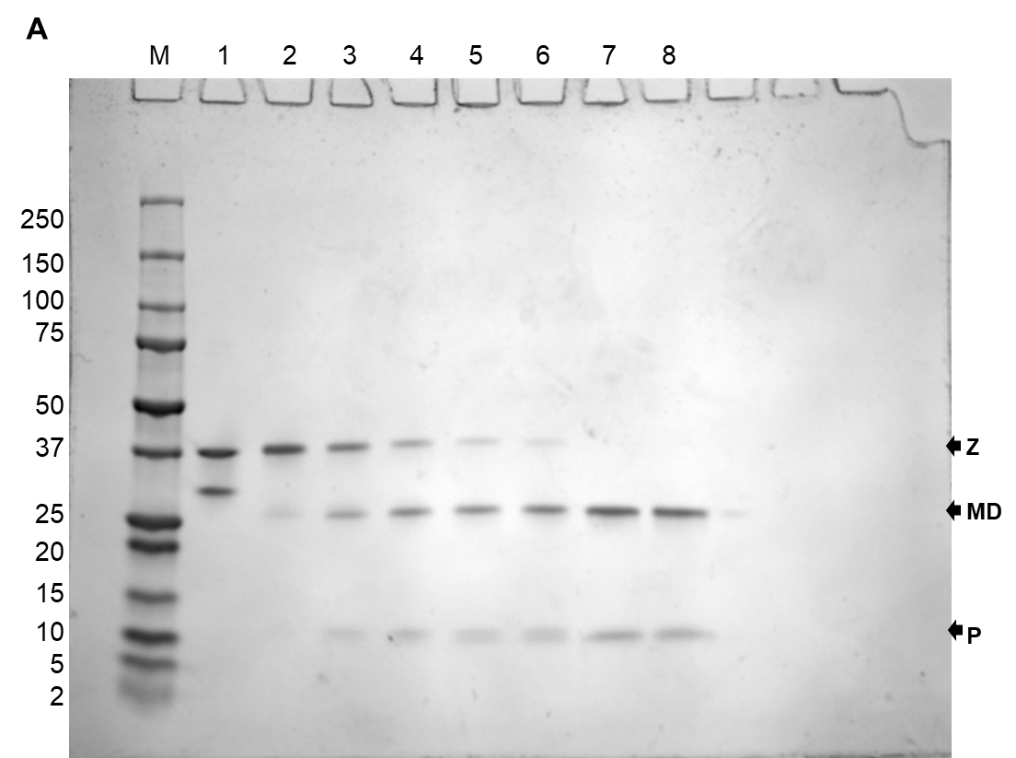


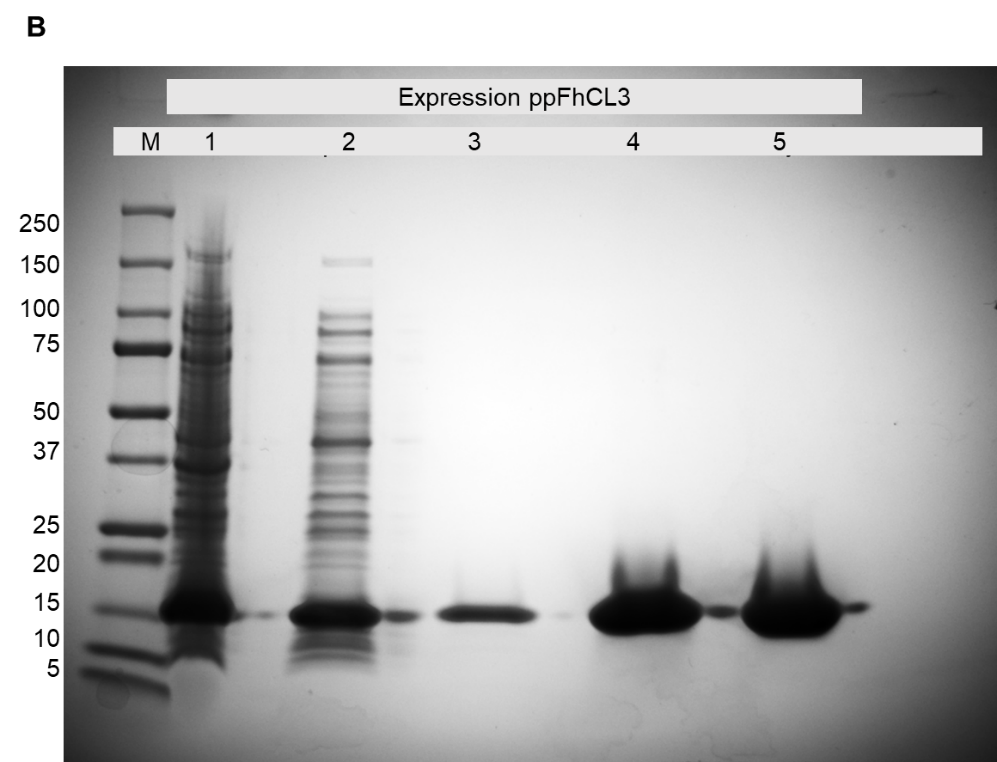


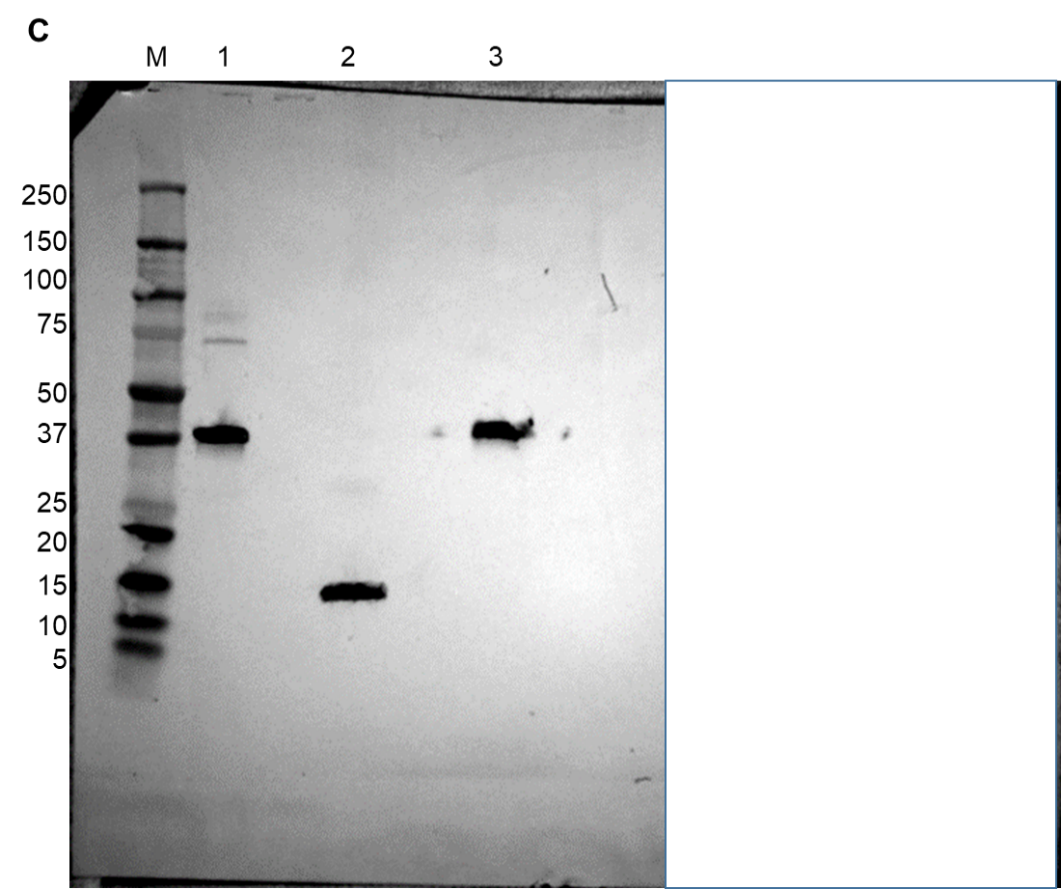


**Additional file 5. Fig S5A.Production of functional recombinant FhCL3 zymogen and FhCL3 propeptide.** (A) SDS-PAGE gel showing the activation of the recombinant FhCL3 zymogen. Lane 1, Control incubation. Recombinant FhCL3 zymogen expressed in the yeast *P. pastoris* was incubated without activation buffer at 37°C for 5 hr. Lane 2, inactivated recombinant FhCL3 zymogen expressed in the yeast *P. pastoris* and purified using affinity chromatography. Lanes 3 to 8, activation of the FhCL3 zymogen was initiated by incubating the enzyme in sodium acetate buffer pH 4.5 at 37°C. The progression of activation was observed by removing samples at each hour, adding the inhibitor E-64 before resolving in a 4-12% SDS-PAGE gel. Z, zymogen; MD, mature domain of FhCL3; P, the released propeptide. (B) SDS-PAGE gel showing expression of the recombinant FhCL3 propeptide in *E. coli* BL21 cells. Lane 1, cell pellet after induction for 3 hr at 30°C; lane 2, supernatant after extraction of the cell pellet; lane 3, wash after recombinant protein binding to the affinity column (Profinia affinity chromatography system); lane 4, the eluted recombinant ppFhCL3; lane 5, the dialysed recombinant ppFhCL3. (C) Western blot analysis of recombinant FhCL3 zymogen (1 µg, lane 1), FhCL3 propeptide (2 µg, lane 2), and somatic extract of NEJs 24 hr (15 µg, lane 3). Immunoblots were probed with anti-ppFhCL3 polyclonal antibodies raised in rabbit. M, Molecular weight in kDa. The white panel denotes proteins not pertinent to this study.


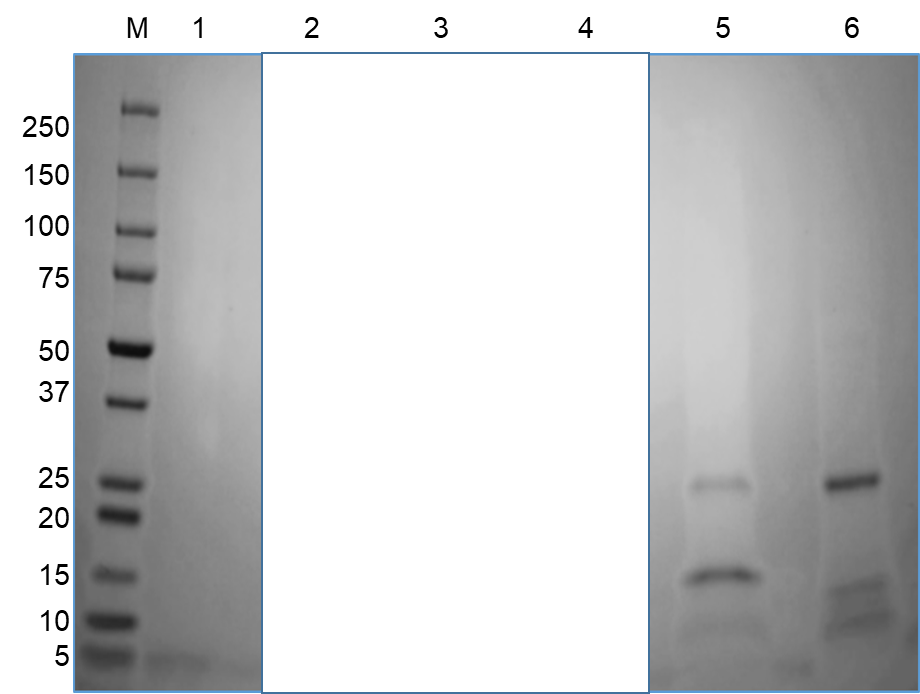


**Additional file 5. Fig S5B. FhCL3 propeptide binds native cathepsin peptidases excreted-secreted by *F. hepatica* adult parasites.** The ppFhCL3 was used to pull-down proteins within the ES proteins from adult *F. hepatica* and the results were analysed by SDS-PAGE gels, as follows: Lane 1, ES proteins from *F. hepatica* adult parasites pulled down with Ni-NTA beads; lanes 2 to 4 coved by the white panel denote proteins not pertinent to this study. Lane 3, ES proteins from *F. hepatica* adult parasites pulled down with Ni-NTA beads/recombinant ppFhCL3; lane 5, ES proteins from *F. hepatica* adult parasite; M, molecular weight in kDa.
