# Supplementary material for: Regulation of the Fasciola hepatica newly excysted juvenile cathepsin L3 (FhCL3) by its propeptide: a proposed ‘clamp-like’ mechanism of binding and inhibition
Source: BMC Mol Cell Biol. 2020 Dec 7;21:90. doi: 10.1186/s12860-020-00335-5 (PMC7720491; doi:10.1186/s12860-020-00335-5)
Supplement: Supplementary file 6 — Additional file 6: Fig. S6A-B. Antibodies against FhCL3 zymogen differentially recognize the recombinant FhCL3 zymogen, mature domain and propeptide segment. [file 12860_2020_335_MOESM6_ESM.docx]

**Additional file 6. Antibodies against FhCL3 zymogen differentially recognize the recombinant FhCL3 zymogen, mature domain and propeptide segment.**


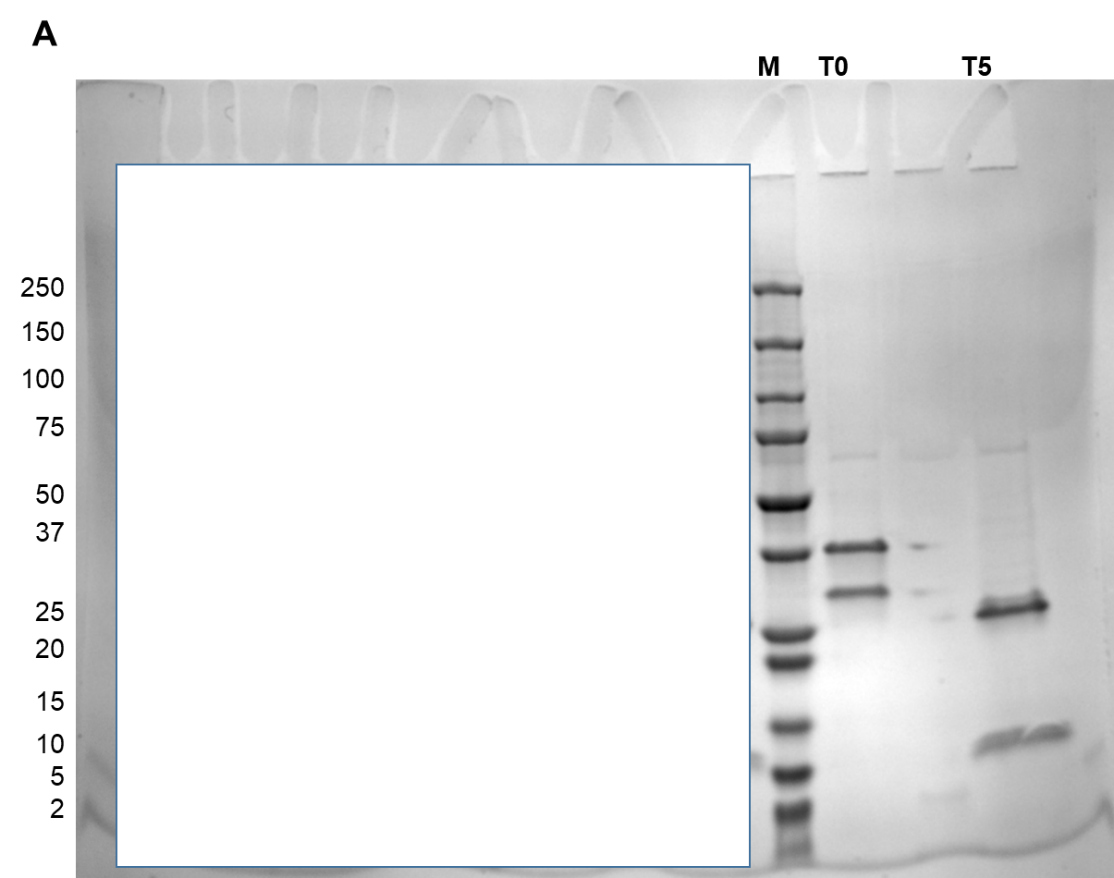


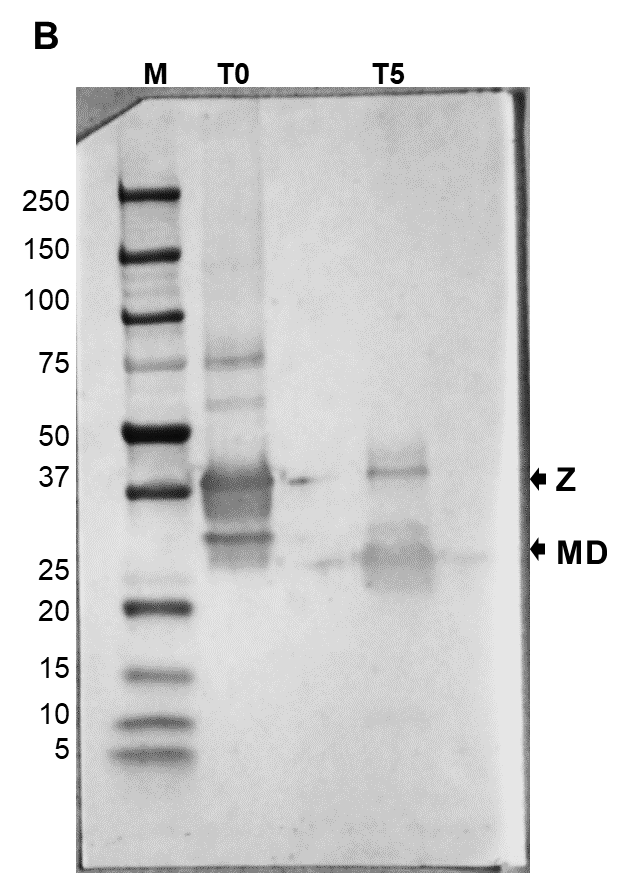


**Additional file 6. Antibodies against FhCL3 zymogen differentially recognize the recombinant FhCL3 zymogen, mature domain and propeptide segment.** (A) SDS-PAGE gel showing the activation of the recombinant FhCL3 zymogen. T0: Inactivated recombinant FhCL3 zymogen expressed in the yeast P. pastoris and purified using affinity chromatography. T5: Activation of the FhCL3 zymogen was initiated by incubating the enzyme in sodium acetate buffer pH 4.5 at 37°C. At time 5hr an aliquot was removed and the inhibitor E-64 was added to the sample before resolving it in a 4-12% SDS-PAGE gel. Z, zymogen (~37 kDa); MD, mature domain of FhCL3 (~25 kDa); P, the released propeptide (~12 kDa). (B) Western blot analysis of the recognition of FhCL3 zymogen, mature domain and propeptide released during peptidase activation by the polyclonal anti-FhCL3 antibodies. Samples of recombinant FhCL3 zymogen (5 µg) or FhCL3 activated 5hr (5 µg) were probed with anti-FhCL3 polyclonal antibodies raised in rabbit (1:15,000). Z, zymogen; MD, mature domain of FhCL3. M, Molecular weight in kDa. Note that the polyclonal response to the FhCL3 is directed towards the mature domain (MD rather than to the propeptides (P).The white panel denotes proteins not pertinent to this study.
